# Guest-induced pore breathing controls the spin state in a cyanido-bridged framework†

**DOI:** 10.1039/d3sc03255h

**Published:** 2023-08-16

**Authors:** Michał Magott, Klaudia Płonka, Barbara Sieklucka, Katarzyna Dziedzic-Kocurek, Wataru Kosaka, Hitoshi Miyasaka, Dawid Pinkowicz

**Affiliations:** a Faculty of Chemistry, Jagiellonian University Gronostajowa 2 30-387 Kraków Poland michal.magott@uj.edu.pl dawid.pinkowicz@uj.edu.pl; b Institute for Materials Research, Tohoku University 2-1-1 Katahira, Aoba-ku Sendai 980-8577 Japan miyasaka@imr.tohoku.ac.jp; c Marian Smoluchowski Institute of Physics, Jagiellonian University Stanisława Łojasiewicza 11 Kraków 30-348 Poland

## Abstract

Iron(ii) spin cross-over (SCO) compounds combine a thermally driven transition from the diamagnetic low-spin (LS) state to the paramagnetic high-spin (HS) state with a distinct change in the crystal lattice volume. Inversely, if the crystal lattice volume was modulated post-synthetically, the spin state of the compound could be tunable, resulting in the inverse effect for SCO. Herein, we demonstrate such a spin-state tuning in a breathing cyanido-bridged porous coordination polymer (PCP), where the volume change resulting from guest-induced gate-opening and -closing directly affects its spin state. We report the synthesis of a three-dimensional coordination framework {[Fe^II^(4-CNpy)_4_]_2_[W^IV^(CN)_8_]·4H_2_O}_*n*_ (1·4H_2_O; 4-CNpy = 4-cyanopyridine), which demonstrates a SCO phenomenon characterized by strong elastic frustration. This leads to a 48 K wide hysteresis loop above 140 K, but below this temperature results in a very gradual and incomplete SCO transition. 1·4H_2_O was activated under mild conditions, producing the nonporous {[Fe^II^(4-CNpy)_4_]_2_[W^IV^(CN)_8_]}_*n*_ (1) *via* a single-crystal-to-single-crystal process involving a 7.3% volume decrease, which shows complete and nonhysteretic SCO at *T*_1/2_ = 93 K. The low-temperature photoswitching behavior in 1 and 1·4H_2_O manifested the characteristic elasticity of the frameworks; 1 can be quantitatively converted into a metastable HS state after 638 nm light irradiation, while the photoactivation of 1·4H_2_O is only partial. Furthermore, nonporous 1 adsorbed CO_2_ molecules in a gated process, leading to {[Fe^II^(4-CNpy)_4_]_2_[W^IV^(CN)_8_]·4CO_2_}_*n*_ (1·4CO_2_), which resulted in a 15% volume increase and stabilization of the HS state in the whole temperature range down to 2 K. The demonstrated post-synthetic guest-exchange employing common gases is an efficient approach for tuning the spin state in breathing SCO-PCPs.

## Abstract 2

Variation of three different stimuli: the type of guest molecules, temperature and light enables intentional manipulation of the mechanical, electronic and magnetic properties of a cyanido-bridged coordination polymer.

## Graphical abstract

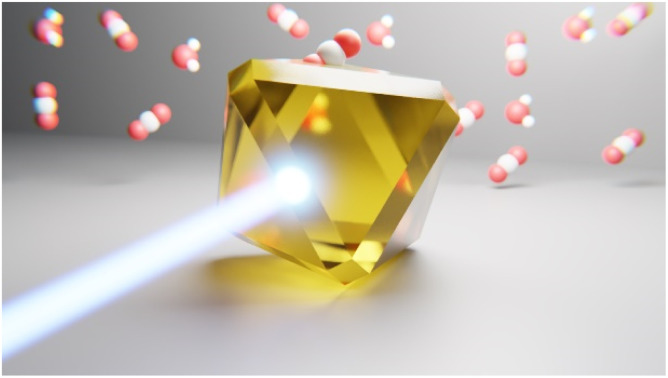

## Introduction

**Fig. 1:**
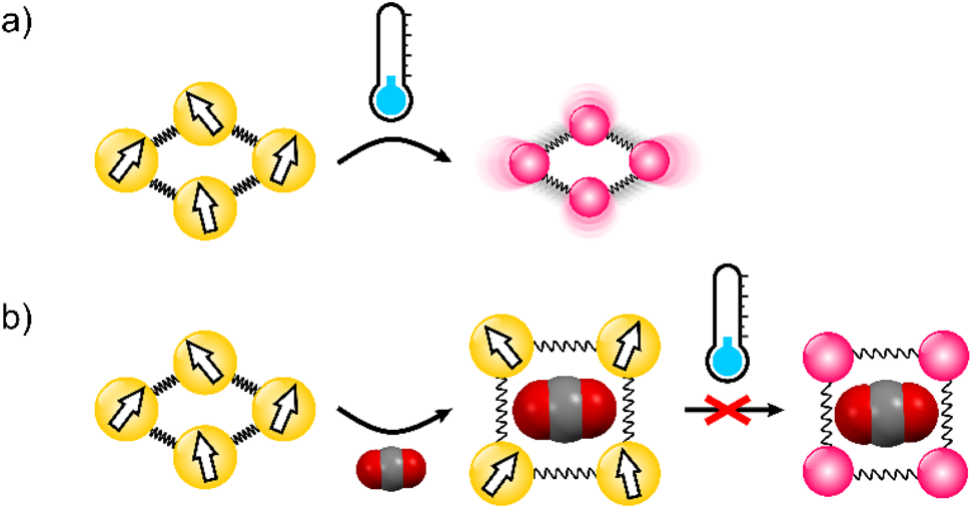
(a) Thermally induced high-spin to low-spin transition is typically associated with lattice contraction. (b) Volume expansion triggered by guest inclusion in the breathing coordination framework prevents spin transition to the small-volume low-spin state.

The spin cross-over (SCO) phenomenon is a transition between low-spin (LS) and high-spin (HS) states of metal ions induced by a physical stimulus such as temperature, pressure, or light irradiation.^1^ For octahedral iron(ii) compounds in the solid state, a transition from the diamagnetic state to the paramagnetic *S* = 2 state is intertwined with the significant expansion of the crystal volume (Fig. 1a), as well as the variation in optical and electric properties.^2–4^ The simultaneous change of multiple properties makes SCO materials desirable for the nanofabrication of single-molecule junctions and thin films sensitive to environmental factors.^5–8^ Although control over the spin state is usually achieved by temperature or mechanically, in their seminal work Kepert *et al.* demonstrated that the SCO properties can also be modulated with the uptake and release of the guest molecule in a porous coordination polymer (PCP).^9^ The field expanded exponentially when Ohba *et al.* presented complete chemo-switching of the spin state in Hofmann-type PCPs,^10,11^ and since then, numerous {Fe^II^(L)[M^II^(CN)_4_]}_*n*_ (M = Ni, Pt) compounds were reported to show the effect of the guest adsorption on the SCO phenomenon.^12^ While SCO frameworks show volume change resulting from the change in the spin state, large variation of crystal lattice volume upon guest uptake is also characteristic of PCPs which demonstrate the so-called “breathing” behavior.^13–16^ As both SCO and guest-induced breathing result in crystal swelling/shrinking, one may expect that combination of these two phenomena would lead to a very strong coupling of these properties, that is, stabilization of the small-volume LS state for the closed-pore phase and the large-volume HS state in the open-pore phase (Fig. 1). We expect that the crystal lattice volume closely associated with the spin state could be tuned by the kind of guest molecules being loaded into pores. This assumption is further supported by the pronounced effect of guest-induced gate-opening on the phase transition observed in donor–acceptor MOFs, reported recently by some of us.^17,18^ Noticeably, this phenomenon is hardly evidenced in rigid three-dimensional (3-D) Hofmann-type PCPs, in which strong host–guest interactions with guest molecules seem to overcome the volume change effect.^19–22^ Thus, in order to successfully observe the aforementioned coupling of the crystal lattice breathing with the spin transition, one should either decrease the strength of specific host–guest interactions or increase the volume change in the breathing SCO framework. The prospective candidate can be found in the family of octacyanidometallate-based compounds {[Fe^II^(py)_4_]_2_[M^IV^(CN)_8_]·*x*H_2_O}_*n*_ (py = pyridine derivatives, M = Mo, W, Nb, Re),^23^ since some of us recently demonstrated large water-induced breathing of the octacyanomolybdate(iv) coordination polymer.^24^ The impact of guest molecules on SCO properties of octacyanidometallate frameworks was mostly overlooked, with the exception of the water-induced spin transition reported by Song *et al.* in {[Fe^II^((3-pyridyl)methanol)_4_]_2_[W^IV^(CN)_8_]·4H_2_O}_*n*_.^25^ Herein, we report {[Fe^II^(4-CNpy)_4_]_2_[W^IV^(CN)_8_]}_*n*_ (1; 4-CNpy = 4-cyanopyridine), a flexible nonporous framework characterized by a weakly cooperative spin transition at *T*_1/2_ = 93 K. Upon adsorption of water molecules, it showed a gate-opening breathing transition with a 7.3% increase of crystal volume resulting in {[Fe^II^(4-CNpy)_4_]_2_[W^IV^(CN)_8_]·4H_2_O}_*n*_ (1·4H_2_O). The reorganization of the crystal framework led to the appearance of a 48 K wide hysteresis loop in the 187–235 K range, accompanied by strong elastic frustration, with the low-temperature phase showing a mixed low-spin/high-spin state. The most striking feature of the nonporous 1 is the ability to accommodate four CO_2_ molecules per formula unit in a gate-opening fashion, leading to {[Fe^II^(4-CNpy)_4_]_2_[W^IV^(CN)_8_]·4CO_2_}_*n*_ (1·4CO_2_). Finally, this CO_2_ uptake resulted in a 15% increase in crystal volume, which in turn stabilized the HS state in the whole temperature range of 2–300 K. Herein, we demonstrate the total control of the spin state of a PCP driven by guest-dependent breathing-induced steric effects.

## Results and discussion

### Crystal structures and water adsorption properties

**Fig. 2:**
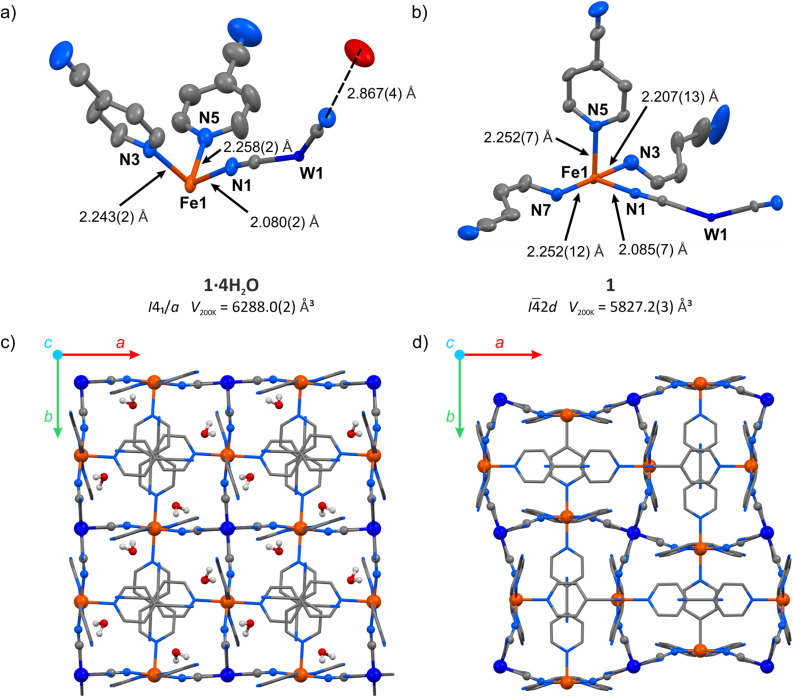
Structural transformation accompanying the transition from 1·4H_2_O to 1: asymmetric unit of 1·4H_2_O (a) and 1 (b) with selected bond distances (hydrogen atoms were omitted for clarity), as well as coordination frameworks of 1·4H_2_O (c) and 1 (d) as seen along the crystallographic *c*-axis (hydrogen atoms of 4-cyanopyridine molecules were omitted for clarity). W – dark blue, Fe – orange, O – red, N – blue, C – grey.

The hydrated compound {[Fe^II^(4-CNpy)_4_]_2_[W^IV^(CN)_8_]·4H_2_O}_*n*_ (1·4H_2_O) was obtained as orange crystals by a wet chemistry approach (see the Experimental for details). It crystalized as a 3-D coordination network in a tetragonal crystal system, space group *I*4_1_/*a* (Table S1†). All Fe(ii) centres in the structure of 1·4H_2_O are surrounded by six nitrogen atoms and equivalent by symmetry. The iron(ii) coordination sphere consists of four nitrogen atoms of aromatic 4-cyanopyridine rings and two cyanido ligands in a trans configuration (Fig. 2a). The observed Fe–N bond lengths at 200 K are 2.080(2) Å for Fe–NC and 2.243(2) or 2.258(2) Å for Fe–N_pyridine_, in line with expectations for iron(ii) in a high-spin (HS) state.^25–34^ Octacyanidotungstate(iv) forms four cyanide bridges with iron(ii), leaving four terminal cyanides engaged in hydrogen bonding with water molecules with a CN⋯O distance of 2.867(5) Å (Table S2†). Crystallization water molecules do not leave empty spaces in the framework (no void spaces were detected for 1.3 Å probe radius,^35^ while the kinetic diameter of water is estimated to be 2.65 Å).^36^ Noteworthily, the water molecules also form O⋯NC contact with the nitrogen atom belonging to the nitrile group of 4-cyanopyridine (3.074(7) Å). Despite the aromatic nature of the pyridine rings, no π–π interactions are observed, since all pyridine molecules are separated by more than 4 Å. However, 4-cyanopyridine molecules interact with each other through strongly polarized nitrile groups, which form –CN⋯C contacts characterized by the 3.244(5) Å intermolecular distance. Cooling the crystal of 1·4H_2_O below 190 K led to an immediate increase in crystal mosaicity and the disappearance of high-resolution (<1.0 Å) diffraction peaks and therefore the crystal structure could not be analysed at lower temperatures.

Thermogravimetric analysis (TGA) performed on a powder sample of 1·4H_2_O under dry nitrogen flow shows an immediate decrease in mass already at room temperature (Fig. S1†). Within four minutes of measurement and below 30 °C, the mass reached a plateau of *m*/*m*_0_ = 96.5%, and the sample mass remained constant up to 100 °C. This value is slightly higher than the expected *m*/*m*_0_ = 94.9% for the completely dehydrated compound, because the dehydration starts immediately after removing the crystals from the mother solution at room temperature. The completeness of water removal is also supported by the powder X-ray diffraction pattern (PXRD) for the sample 1·4H_2_O activated in a vacuum at room temperature, which does not show reflections of the parent 1·4H_2_O phase (Fig. S2†).

Following the TGA results, the single crystal of the hydrated framework 1·4H_2_O was dehydrated *in situ* by purging with a dry nitrogen gas atmosphere at 343 K. The resulting anhydrous phase {[Fe^II^(4-CNpy)_4_]_2_[W^IV^(CN)_8_]}_*n*_ (1) was cooled and the sc-XRD measurement was performed at 200 K. Upon dehydration, the symmetry changes from *I*4_1_/*a* in 1·4H_2_O to *I*4̄2*d* in 1 and the unit cell volume is reduced by 7.3%. In the dehydrated framework, there are four inequivalent nitrogen atoms coordinated to the Fe(ii) centre (Fig. 2b), compared to three different Fe–N bonds in the hydrated form. The corresponding bond lengths are 2.085(7) Å for Fe–NC, and 2.207(13), 2.252(7) and 2.252(12) Å for Fe–N_pyridine_, suggesting a fully HS state of Fe(ii) at 200 K, similarly to the framework before dehydration. Importantly, as a result of water removal, the cyanido bridges underwent distinct bending, with the N–C–Fe angle changing from 170.8(2)° to 162.4(7)°. On the other hand, the equatorial pyridine coordination became more ordered, as N_pyridine1_–Fe–N_pyridine2_ angles change from 87.73(9)° and 92.27(9)° in 1·4H_2_O to 90.0(2)° in 1. The aforementioned changes lead to the reorganization of the coordination skeleton as visible along the *c*-axis, which is depicted in Fig. 2c and d, with very little change observed along the *a* axis (Fig. S3†). The selected bond lengths and angles in structures of 1·4H_2_O and 1 are summarized in Table S2.† After complete evacuation of water guest molecules, the anhydrous 1 shows no cavities for the 1.3 Å radius probe molecule. This points to a breathing phenomenon, which can be described as a gate-closing process.^16,37^ Although gate-opening (GO) and gate-closing (GC) processes were recently demonstrated in amorphous^38^ and microcrystalline^24,39,40^ cyanido-bridged frameworks, to our knowledge the transition from 1·4H_2_O to 1 constitutes the first example of gate-closing visualized by single-crystal XRD in a coordination polymer featuring only cyanido bridges.

**Fig. 3:**
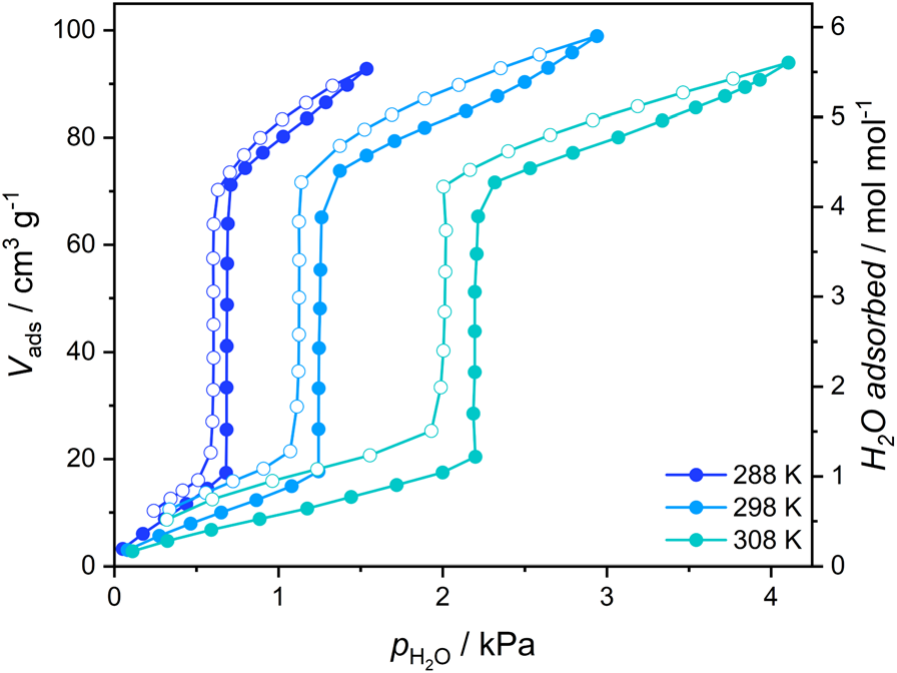
Vapor adsorption isotherms of water recorded for 1 (1·4H_2_O activated in a vacuum at 323 K). Full circles – adsorption, open circles – desorption.

The gate-opening/closing character of the 1 ↔ 1·4H_2_O transformation is further confirmed by the measurement of volumetric adsorption of water vapor for 1 at 288–308 K (Fig. 3). Adsorption curves up to the threshold water pressure can be classified as type III isotherms typical for nonporous materials, with approximately one water molecule per Fe_2_W formula unit being adsorbed. However, a sudden increase in the amount of adsorbed vapor characteristic of GO can be observed above 40% relative humidity and the adsorbed volume after step-like increase corresponds to *ca.* 4.25 H_2_O, almost perfectly in line with {[Fe^II^(4-CNpy)_4_]_2_[W^IV^(CN)_8_]·4H_2_O}_*n*_ formulation deduced for 1·4H_2_O from sc-XRD measurement. A small further increase in adsorption occurs at higher vapor pressures, which probably results from water adsorption on the surface of crystallites. In the desorption branch, the adsorbed amount of water slowly decreases until it reaches 4.25 mol mol^−1^ around 35% relative humidity. Below this pressure, an abrupt decrease in the amount of adsorbed vapor corresponding to the GC process can be observed.

The pronounced structural transformation that accompanies the gate-opening process leads to several changes in the infrared (IR) spectrum of 1 (Fig. S4a†). Water adsorption is accompanied by the appearance of a broad O–H stretching band in the 2800–3700 cm^−1^ range and the H–O–H bending band at 1629 cm^−1^, both confirming the presence of water molecules engaged in hydrogen bonding with cyanide ligands in the structure of 1·4H_2_O. Furthermore, the nitrile stretching bands of 4-cyanopyridine molecules change their structure (Fig. S4b†), with two unequal components at 2237 and 2241 cm^−1^ in the case of 1 reaching equal intensities at 2240 and 2247 cm^−1^ for 1·4H_2_O. This is correlated with three symmetrically independent 4-cyanopyridine molecules in the structure of 1, as opposed to only two in 1·4H_2_O. All other 4-cyanopyridine vibrations in the fingerprint region shift by 2–3 cm^−1^ toward higher energies upon water adsorption, which is interpreted as general stiffening of the coordination skeleton.

### Magnetic properties of 1·4H_2_O and 1

**Fig. 4:**
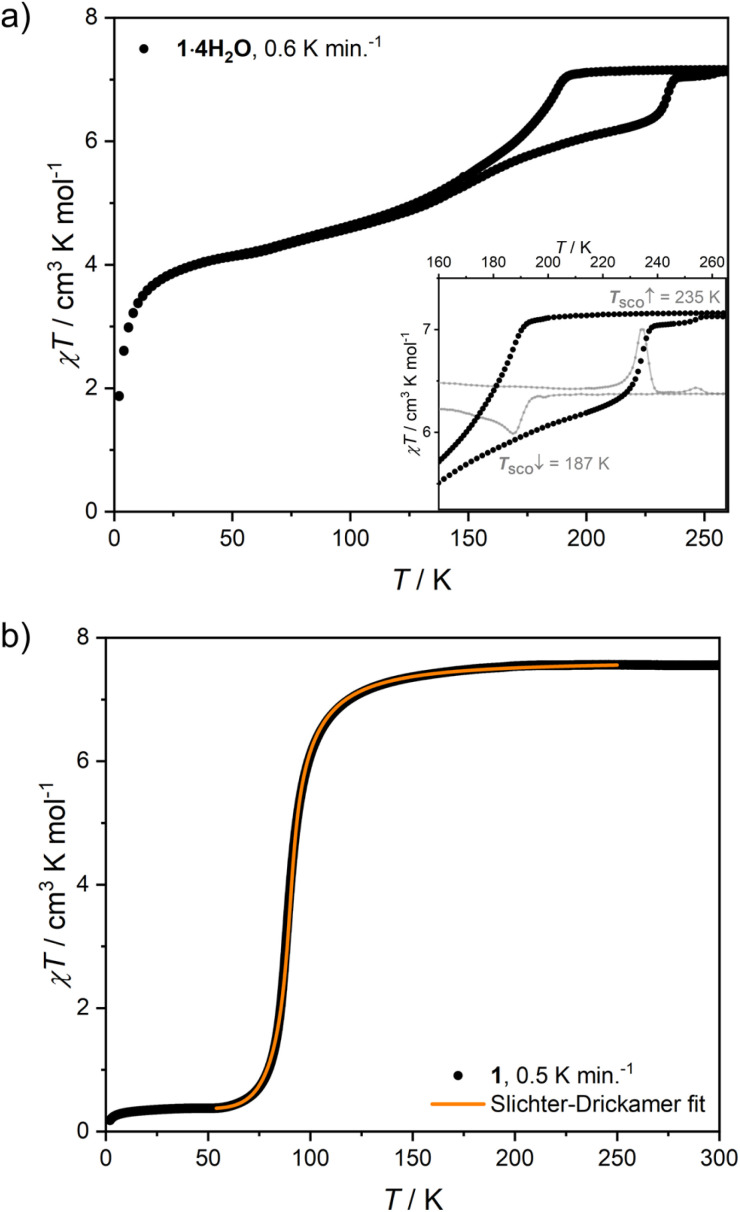
*χT*(*T*) dependence recorded for 1·4H_2_O at *μ*_0_*H*_dc_ = 0.1 T (a) and close-up of the hysteresis loop with the corresponding d(*χT*)/d*T* derivative (inset), *χT*(*T*) dependence recorded for 1 at *μ*_0_*H*_dc_ = 0.1 T and the fit of the Slichter–Drickamer model (Eq. (2)) to the experimental data (b).

Structural data at 200 K are consistent with both 1·4H_2_O and 1 being composed of paramagnetic Fe(ii) ions (*S* = 2). Consequently, 1·4H_2_O is characterized by magnetic susceptibility and temperature product (*χT*) of 7.16(11) cm^3^ K mol^−1^ at 265 K (Fig. 4a), which corresponds to two high-spin iron(ii) centres per Fe_2_W formula unit and the Landé factor *g*_Fe_ = 2.18(2). A relatively fast decrease of *χT* was observed below 192 K, reaching 5.23 cm^3^ K mol^−1^ at 140 K. Below this point, a decrease of *χT* was very gradual, reaching 4.19 cm^3^ K mol^−1^ at 60 K (58.5% of the room temperature value). All changes of *χT* product in the 192–60 K range are attributed to the spin cross-over phenomenon, which is confirmed by Mössbauer spectroscopy measurements performed on cooling (Fig. S5 and Table S3†). A further decrease of *χT* below 60 K is unlikely to result from SCO and is expected to originate from the zero-field splitting effect and/or antiferromagnetic dipole–dipole interactions. The *χT* thermal dependence is reversible on heating to 140 K, when it starts to diverge from that recorded on cooling. The discrepancy of the cooling and heating processes manifests itself as a thermal hysteresis of SCO, with *T*_SCO_ (defined as a maximum of the d(*χT*)/d*T* derivative) equal to 187 K on cooling and 235 K on heating (Fig. 4a, inset). The presence of the hysteresis loop was confirmed by Mössbauer spectroscopy (Fig. S5†) and dynamic scanning calorimetry (DSC; Fig. S6†). The simultaneous presence of a wide hysteresis loop above 180 K and an incomplete gradual transition below 140 K must result from elastic frustration of the framework.^41,42^ Because of the hysteretic behaviour accounting only for 0.25–0.3 mol Fe per Fe_2_W formula unit, as well as the rather blurred nature of the transition at 187 and 235 K, the signal intensity observed in the DSC measurement is relatively weak (Fig. S6a and b†). Because of that, the enthalpy of the transformation was determined only for the transition on heating. Depending on the background subtraction procedure (Fig. S6c†) and the assumed amount of SCO-active centres (0.25–0.3 per Fe_2_W), the resulting transformation enthalpy is Δ*H*_SCO_ = 8.5–11.4 kJ mol^−1^ Fe, leading to Δ*S*_SCO_ = 38–51 J K^−1^ mol^−1^.

Similarly to 1·4H_2_O, room temperature *χT* product observed for 1 equals 7.56(18) cm^3^ K mol^−1^ (Fig. 4b), as expected for two centres of high-spin iron(ii) with *g*_Fe_ = 2.24(3) cm^3^ K mol^−1^. No change in *χT* is observed down to *ca.* 150 K, below which point the gradual decrease starts. Below 100 K a fast decrease occurs reaching 0.38 cm^3^ K mol^−1^ at 50 K. The observed change represents 95% of the room temperature signal and is interpreted as a complete SCO transition, in line with the results of Mössbauer spectroscopy (Fig. S7 and Table S4†), which shows a pure HS state at 150 K and a pure LS state at 62 K. This points towards a weakly cooperative SCO transition that can be quantified with the Slichter–Drickamer model:^43^1
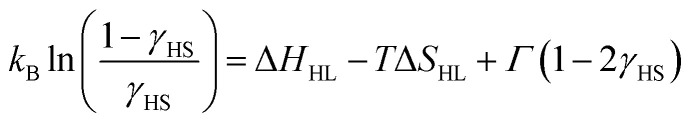


Fitting the experimental *χT* curve in the 50–250 K range yields the following parameters: Δ*H*_HL_ = 4.74(3) kJ mol^−1^, Δ*S*_HL_ = 51.0(3) J mol^−1^ K^−1^ and *Γ* = 1.12(1) kJ mol^−1^ with *R*^2^ = 0.99999. The determined SCO temperature *T*_SCO_ = Δ*H*_HL_/Δ*S*_HL_ = 93(1) K is very low, which for the Δ*S*_HL_ in the typical range (40–80 J K^−1^ mol^−1^)^44^ must result from small Δ*H*_HL_. Typically, SCO compounds that demonstrate a spin transition below 100 K become kinetically trapped in the HS state due to the slow dynamics of the spin transition.^45–48^ However, 1 demonstrates exactly the same SCO behaviour also for a higher temperature sweep rate of 2 K min^−1^ (Fig. S8†). To further study the observed transition, we performed additional diffraction measurements at 140 K and 80 K. When 1 is cooled by 60 K from 200 K to 140 K, Fe–N bonds are shortened by 0.001–0.036 Å (on average 0.017(12) Å) for 4-cyanopyridine ligands and by 0.003 Å for nitrogen atoms belonging to cyanido ligands, which is mostly attributed to the thermal expansion. On the other hand, cooling by another 60 K down to 80 K leads to the much more distinct change of 0.112–0.221 Å (on average 0.17(4) Å) for 4-cyanopyridine and 0.125(7) Å for cyanido ligands, respectively. The sudden decrease of the bond length in the 140–80 K temperature range is in line with the transition temperature determined from magnetic and Mössbauer measurements, and the variation of the bond length is consistent with observations for other SCO frameworks featuring [Fe^II^(py)_4_(μ-NC)_2_] moieties.^25–34^ As the observed contraction of bond lengths falls in the typical range for similar assemblies, this raises the question about the source of unusually small Δ*H*_HL_. This could be explained by stabilization of the HS state or destabilization of the LS state. No such effect can be easily identified by comparison of intermolecular interactions in the structures of 1 at 140 and 80 K (Table S2†). Therefore, we hypothesize that a small value of Δ*H*_HL_ may result from the destabilizing effect of intermolecular repulsion in the low-spin state of 1, which is expected to be sterically crowded, since the expanded high-spin state lacks void spaces.

Although both 1·4H_2_O and 1 share the same connectivity of the coordination skeleton, the above-described SCO behaviour of these two phases is very different. On the one side, 1·4H_2_O shows a 48 K wide hysteresis loop, unprecedented among octacyanidometallate-based SCO frameworks.^25–34,49^ This points to high cooperativity of the system, but is accompanied by a relatively gradual *χT* change in the 60–140 K range. Increasing the temperature sweep rate does not lead to a significant change in the width of the hysteresis loop (Fig. S9†), yet it decreases the total amount of iron(ii) centres that undergo the SCO phenomenon. This suggests relatively slow kinetics of hysteretic SCO for 1·4H_2_O around 150 K, compared to the fast and gradual SCO observed for 1 below 100 K, leading us to two conclusions. First, given the slightly cooperative nature of SCO in 1, which comprises only [–NC–W^IV^(CN)_6_–CN–] bridges as elastic interaction pathways, elastic frustration of 1·4H_2_O must arise from the additional source of antiferroelastic interactions.^50^ Most likely the appearance of hydrogen bonds increases cooperativity of the spin transition, but water molecules themselves act as stiff rods separating iron(ii) centres. Taking into account the lack of free void spaces in the nonporous 1, which accommodates water vapor only in the pore-opening transition, the steric effect of H_2_O molecules must prevent the contraction of the framework to the full LS state. As a result, SCO for 1·4H_2_O is incomplete (representing approximately half iron(ii) centres) at low temperatures. On the other side, the unusually fast kinetics of the SCO transition observed for 1 below 100 K suggests a high degree of structural flexibility, which is confirmed by observation of the pore-opening behaviour induced by water vapor.

### Gas adsorption studies for 1

**Fig. 5:**
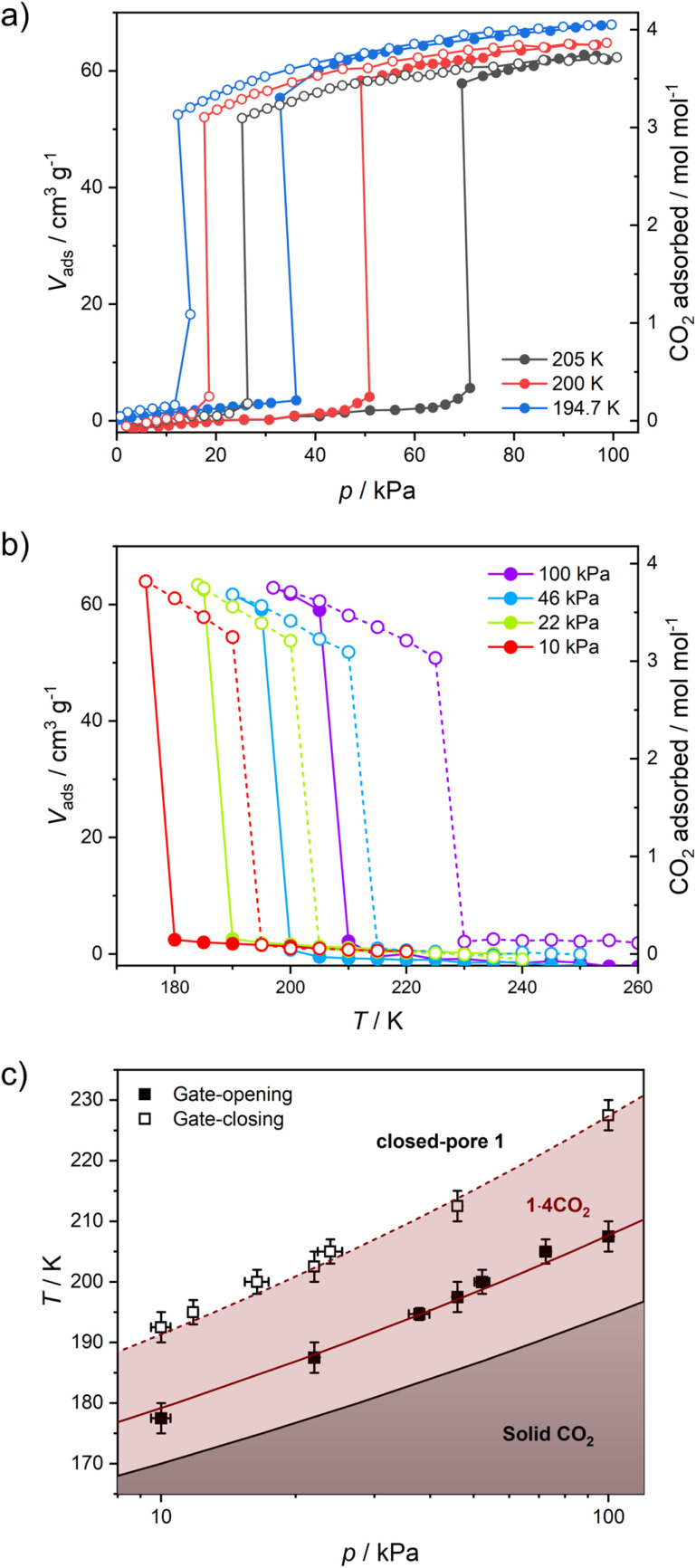
CO_2_ adsorption in 1 studied as isotherms at different temperatures (a) and isobars at different CO_2_ pressures (b), and phase diagram of the 1/CO_2_/1·4CO_2_ system based on these results (c).

Structural flexibility deduced for 1 from magnetic and vapor adsorption studies encouraged us to test its adsorption capability with other gas molecules. However, no gas adsorption was observed for N_2_ and CO at 77 K, as well as NO at 121 K (Fig. S10†). On the other hand, at *T* ≥ 195 K, 1 shows stepped CO_2_ adsorption (Fig. 5a). The transition between the nonporous and open-pore phase is almost binary, since no more than 5 cm^3^ g^−1^ of carbon dioxide is adsorbed below the gate-opening pressure. The total volume of adsorbed CO_2_ at 100 kPa reaches 62–68 cm^3^ g^−1^ (in 205–194.7 K range), which corresponds to approximately 4 CO_2_ molecules per Fe_2_W formula unit. This amount is also reflected in the isobar measurements of CO_2_ adsorption depicted in Fig. 5b. Thus, the formulation deduced for the CO_2_-adsorbed phase from adsorption experiments is {[Fe^II^(4-CNpy)_4_]_2_[W^IV^(CN)_8_]·4CO_2_}_*n*_ (1·4CO_2_), very similar to the composition observed for 1·4H_2_O from sc-XRD. Powder X-ray diffraction measurements performed *in situ* under 100 kPa CO_2_ on cooling show the disappearance of the parent phase 1 and the appearance of a new phase below 215 K (Fig. S11†), in line with the gas adsorption isobars. Similarly, 1·4CO_2_ starts to disappear at 230 K when heated, with concomitant restoration of 1. This proves that only one crystal phase of 1·4CO_2_ exists in the 200–230 K range. The reversibility of the transition was also tested by PXRD under isothermal conditions, with the powder pattern typical for 1 being restored by vacuum pumping of 1·4CO_2_ (Fig. S12†). In order to determine thermodynamic parameters, pressure-temperature points for a gate-opening and a gate-closing process determined from isotherms and isobars were fitted to the Clausius–Clapeyron relationship (Fig. S13†):2
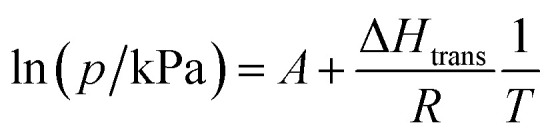


This approach results in *A*_GO_ = 19.1(5), Δ*H*_GO_ = −25.0(8) kJ mol^−1^ and *A*_GC_ = 16.8(4), Δ*H*_GC_ = −23.0(7) kJ mol^−1^. These parameters were used to prepare the phase diagram of the 1/CO_2_/1·4CO_2_ system presented in Fig. 5c.

The powder X-ray diffraction pattern observed for 1·4CO_2_ resembles the powder pattern of 1·4H_2_O, although with two apparent differences (Fig. S14†). All reflections are shifted towards lower *2θ* values, suggesting even bigger expansion of the crystal lattice upon CO_2_ adsorption as compared to the adsorption of H_2_O. Furthermore, even though the powder patterns for 1·4CO_2_ and 1·4H_2_O seem similar, many additional reflections are observed for the former (even after neglecting the remnant reflections of phase 1, depicted in Fig. S12†). The unit cell search and the Le Bail refinement show good agreement for the orthorhombic cell (*a* = 20.364(3) Å, *b* = 19.950(3) Å, *c* = 16.461(3) Å with *R*_wp_ = 1.83%, Fig. S15†). Although these parameters are similar to the tetragonal cell observed for 1 at 200 K (Table S1†), the expansion of the unit cell is observed in all the crystallographic directions and is anisotropic (Δ*a*/*a* = 6.1%, Δ*b*/*b* = 3.9% and Δ*c*/*c* = 4.1%). The total 14.8% volume expansion from 1 (*V*_200K_ = 5827.2(3) Å^3^) to 1·4CO_2_ (*V*_200K_ = 6687(3) Å^3^), corresponds to 860 Å^3^. For *Z* = 4 (as observed in 1 and 1·4H_2_O), this is in line with {[Fe^II^(4-CNpy)_4_]_2_[W^IV^(CN)_8_]·4CO_2_}_*n*_ formulation, as 16 CO_2_ molecules per formula unit are expected to occupy ≈744 Å^3^.

### *In situ* CO_2_ adsorption IR and Raman studies

In order to better understand the host–guest interactions in the structure of 1·4CO_2_, we performed *in situ* IR and Raman spectroscopy studies under a CO_2_ atmosphere. Under *in situ* IR measurement conditions the octacyanidotungstate(iv) cyanide stretching mode is well visible (Fig. S16†). The small shift of this transition (≈4 cm^−1^) after gate-opening is hard to explain on its own, while the very strong bands of gaseous CO_2_ obscure the nitrile stretching bands of 4-cyanopyridine molecules. This issue is resolved by Raman spectroscopy measurement, in which no signal of gaseous CO_2_ is observed in the 2300–2000 cm^−1^ range (Fig. S17†). The single broad peak of 4-cyanopyridine's nitrile can be observed in the Raman spectrum for 1 at 2239 cm^−1^, which after CO_2_ adsorption shifts to 2245 cm^−1^ in 1·4CO_2_. A similar change was observed in the IR spectrum of 1 after H_2_O adsorption (Fig. S4b†), despite the fact that the water molecule only weakly interacts with the nitrile group – the 3.074(7) Å O⋯NC distance in the structure of 1·4H_2_O is 0.13 Å longer than typically observed for hydrogen bonds involving nitriles.^51^ Therefore, the lack of more significant shifts in the Raman spectrum of 1·4CO_2_ suggests that CO_2_ molecules are not involved in any stronger interactions in this structure.

### *In situ* CO_2_ adsorption magnetic studies

**Fig. 6:**
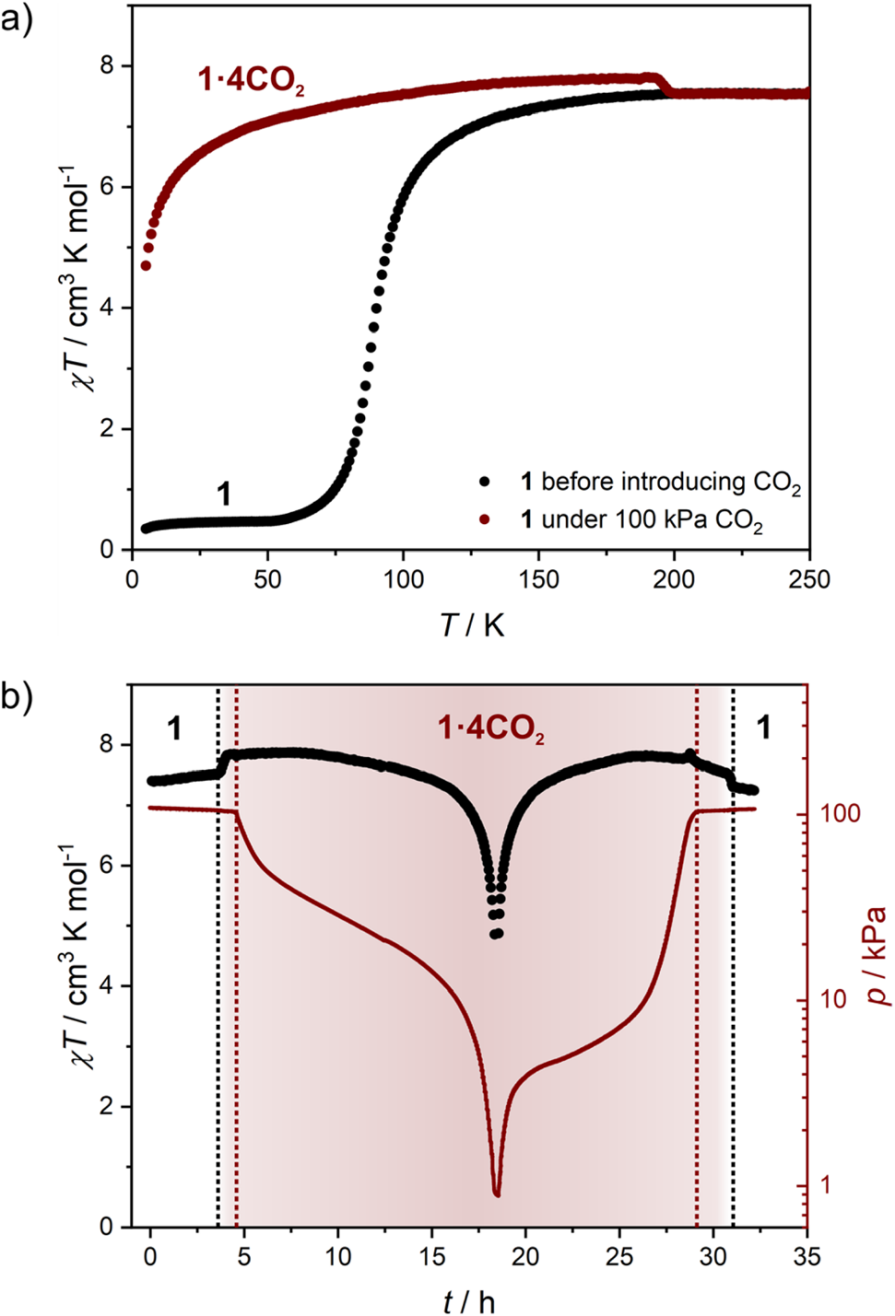
Magnetic studies of 1 performed during *in situ* CO_2_ adsorption: thermal dependence of *χT* for 1 under a 100 kPa He atmosphere (black points) and under a 100 kPa CO_2_ + 5 kPa He atmosphere (dark red line; average cooling rate 0.23 K min^−1^) (a), as well as time dependence of *χT* (black points) and total pressure (dark red line) (b). The zero point in figure (b) corresponds to the start of the measurement depicted with dark red points in figure (a). Dotted lines represent the CO_2_ adsorption/desorption effect (black) and CO_2_ resublimation/sublimation (dark red).

The variation in magnetic properties of 1 upon *in situ* CO_2_ adsorption was tested using a home-built gas cell (see the Experimental for details). A sample of 1·4H_2_O was activated inside the magnetometer chamber and the gas cell was filled with 100 kPa He to facilitate good thermal contact. Then magnetic susceptibility was studied in the 250-5 K range (Fig. 6a, black curve), which perfectly reproduces the behavior of the anhydrous 1 presented in Fig. 4b. Subsequently, the sample was heated to room temperature, and the He pressure was reduced to 5 kPa (to maintain thermal conductivity at low temperatures) and 100 kPa CO_2_ was introduced. This was followed by sample cooling to 250 K, and the magnetic susceptibility was studied again with an average temperature sweep rate of 0.23 K min^−1^. In the 250–200 K range, the behavior of 1 is retained (Fig. 6a, dark red curve), but when cooled from 200 K to 193 K the *χT* increases from 7.54 to 7.80 cm^3^ K mol^−1^. This is a direct result of the CO_2_ adsorption and conversion of 1 to 1·4CO_2_, as evidenced in Fig. 5b, which demonstrates the pressure in the system that was studied in real time. The stepwise *χT* increase induced by CO_2_ adsorption is related to the small decrease in pressure (approximately 1.4 kPa), while the CO_2_ resublimation that starts below 188 K results in a large continuous drop of pressure. The observed temperature range of adsorption in the magnetic measurement is downshifted by ≈10 K as compared to the isobar adsorption measurement under 100 kPa CO_2_ (Fig. 5b, dark red line), which is the result of the slow kinetics of the gate-opening process. The appearance of this 3.4% increase of *χT* is expected to result from the variation in *g*_Fe_ resulting from the CO_2_-induced framework expansion. The adsorption-related nature of this effect was additionally confirmed by the isothermal CO_2_ introduction experiment at 195 K (Fig. S18†), in which no change in magnetic susceptibility is observed upon a fast pressure increase below 40 kPa CO_2_, but a large variation of magnetization begins when the pressure increases above 50 kPa (in line with the phase diagram depicted in Fig. 5c).

Cooling 1·4CO_2_ below 190 K results in only a very small change of *χT* (Fig. 6a, dark red curve). The further decrease observed below 50 K is expected to result from antiferromagnetic interactions between high-spin iron(ii) centers and the zero-field splitting effect. In order to test the reversibility of the magnetic switching behavior, the sample was activated again at 323 K and the experiment was repeated under 50 kPa CO_2_ (Fig. S19†). The behavior of the sample under reduced pressure closely resembles that recorded under 100 kPa CO_2_, but the transformation of 1 into 1·4CO_2_ happens below 198 K instead of 200 K. Phase 1·4CO_2_ under 50 kPa CO_2_ was additionally characterized by the magnetization field dependence at several temperatures, and both *χT*(*T*) and *M*(*H*) curves can be satisfactorily described with the following Hamiltonian constructed in the PHI software^52^ (Fig. S20†):3
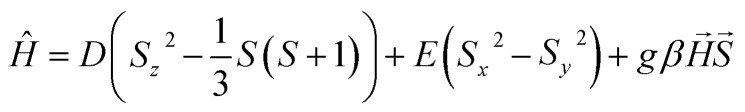


The experimental curves are well reproduced by the following set of parameters, assuming all iron(ii) centers are in the high-spin state: *g* = 2.27(5), *D* = 9.0(1) cm^−1^, *E* = 2.3(1) cm^−1^, with the additional intermolecular interaction parameter *zJ* = −0.085(1) cm^−1^. The obtained values are within the reasonable range for a HS iron(ii),^53–56^ which clearly confirms that the magnetic behavior of 1·4CO_2_ can be properly described without assuming any degree of spin cross-over.

The characterization described above led us to the conclusion that the adsorption of carbon dioxide in 1 quenches SCO and stabilizes the HS state in the whole 2–200 K temperature range. This type of behavior is very rare among iron(ii) spin cross-over compounds, and as far as we know was never observed with CO_2_ as guest molecules. Although CO_2_ adsorption in SCO compounds was previously reported in the literature, it was found to have negligible impact on the magnetic properties of Hofmann-type frameworks^10,11^ or lead to a small modulation of spin transition temperature in other compounds.^57,58^ The only exception was observed recently by Hayami *et al.* in a monomeric Co^II^ compound, where CO_2_ adsorption stabilizes the LS state by strong intermolecular interactions.^59^ On the other hand, the complete stabilization of the HS state in 1·4CO_2_ is expected to originate from the large volume expansion triggered by CO_2_-induced gate-opening (see Table 1). As deduced from X-ray diffraction, the introduction of H_2_O into closed-pore 1 leads to the 7.9% volume expansion, while for CO_2_ this effect is almost doubled with a 14.8% total volume increase. We speculate that this introduces the effect of “internal pressure” on iron(ii) sites, with CO_2_ molecules acting as long rigid rods, preventing network contraction associated with the spin transition. As such, this effect should not be restricted to CO_2_ only, but would also be expected for other gas molecules adsorbed in the breathing frameworks. Although we did not observe breathing behavior for 1 under a N_2_, CO or NO atmosphere (Fig. S10†), we believe that this should inspire the search for other breathing SCO frameworks showing large volume change, as such frameworks could be utilized as highly selective sensors.

**Table 1:** Summary of SCO compounds showing CO_2_ adsorption

Compound	Type	Porosity	CO_2_ adsorbed/mol mol^−1^ Fe	Δ*V*/*V*	Impact on the SCO	Ref.
Fe(pz)[Pt(CN)_4_]	2-D	Microporous	1.1	n/a	None	10
Fe(pz)[Ni(CN)_4_]	2-D	Microporous	0.9	n/a	None	11
[Fe(btzx)_3_](ClO_4_)_2_	1-D	Microporous	0.9	<1%^a^	Δ*T*_1/2_ = +9 K [LS stabilized]	57
[Fe(tpmd)(NCBH_3_)_2_]	3-D	Microporous	0.75–2.5	0.26%	Δ*T*_1/2_ = −29 K [HS stabilized]	58
[Co(COO-terpy)_2_]	0-D	Microporous/gate-opening	1.2–2.0	1.6%	Δ*T*_1/2_ ≈ +100 K^b^ [LS stabilized]	59
[Fe(4-CNpy)_4_]_2_[W(CN)_8_] (1)	3-D	Nonporous/gate-opening	2.0	15%	HS only	This work

a 0.06% volume difference between the CO_2_-adsorbed phase studied at RT and the activated phase studied at 240(2) K.

b Impossible to determine the accurate value due to the very broad transition for the activated phase.

### Photomagnetic properties of 1 and 1·4H_2_O

**Fig. 7:**
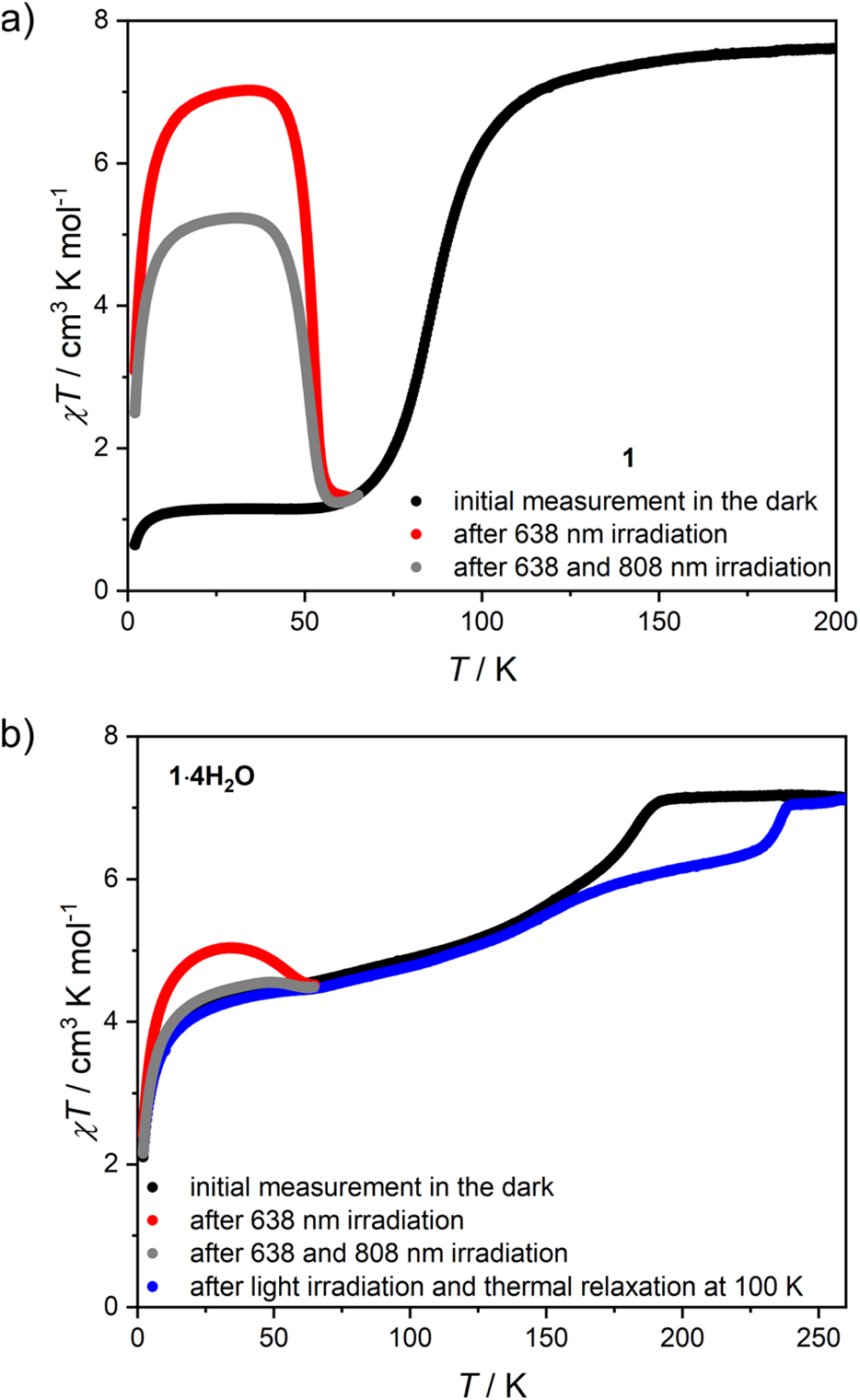
*χT*(*T*) variation recorded after 638 and 808 nm light irradiation for 1 (a) and 1·4H_2_O (b). Initial measurements and measurement after thermal relaxation were performed at 2 K min^−1^ sweep rate, while measurements after light irradiation were performed at 0.3 K min^−1^.

The low-temperature elasticity of 1 and 1·4H_2_O was additionally tested by checking the possibility of inducing a high-spin state with visible light irradiation, the so-called light-induced excited spin state trapping (LIESST) effect.^60^ To allow for efficient irradiation and preserve the anhydrous state in the magnetometer chamber, the polycrystalline sample of 1 was crushed in an agate mortar and sealed inside a polyethylene bag (all operations were carried out under an argon atmosphere). The as-prepared sample reaches a slightly higher value of 1.15 cm^3^ K mol^−1^ at 30 K, compared to 0.38 cm^3^ K mol^−1^ in bulk (Fig. 7a, black line). This may result from either an increase in the number of defects after crystal crushing or from a partial sample decomposition during sealing the polyethylene bag with an impulse heat sealer. However, the phase identity as 1 is confirmed by the appearance of the same *T*_SCO_ as in bulk. Sample irradiation with *λ* = 638 nm at 10 K leads to a fast increase in magnetization, which saturates after 120 minutes (Fig. S21†). After turning off the light and sample thermalization back to 10 K (since constant light irradiation heats the sample by 2–3 K), the *χT* stabilizes at 6.28 cm^3^ K mol^−1^ and shows no evolution in 20 minutes. This was followed by cooling the sample down to 2 K and *χT* measurement at the heating rate of 0.3 K min^−1^ (in order to accurately determine *T*_LIESST_),^61,62^ as demonstrated in Fig. 7a (red line). The *χT* product reaches a maximum of 7.02 cm^3^ K mol^−1^ at 35 K, which is very close to the 7.56 cm^3^ K mol^−1^ observed at room temperature and therefore the LIESST for 1 is concluded to be almost quantitative. Furthermore, the *T*_LIESST_ determined for 1 as an extremum of d(*χT*)/d*T* equals 53 K (Fig. S22†), which in accordance with the formula proposed by Létard *et al.*^63^ yields *T*_0_ = 82 K. Despite the 3-D nature of the coordination skeleton in 1, the *T*_0_ value would classify it among iron(ii) centers surrounded by six independent ligands. Both of these characteristics – the completeness of the photo-induced transition to the HS state and the low *T*_0_ value – further confirm the high degree of structural flexibility in 1. Moreover, the photoinduced HS state in 1 at 10 K is rather persistent, as in the repeated experiment in which 638 nm was followed by 808 nm irradiation (Fig. S23† and 7a, gray line), the photo-induced *χT* was only decreased from 6.00 to 4.70 cm^3^ K mol^−1^. Therefore, the reverse-LIESST effect^64^ triggered by *λ* = 808 nm light allows for deexcitation of only ≈25% metastable HS centers in 1.

Phase 1·4H_2_O was prepared for photomagnetic measurements in a similar way to 1, by sealing crushed crystals inside a polyethylene bag, but with a very small amount of the mother solution, which allows for a perfect reproduction of the magnetic characteristics of the bulk sample (Fig. 7b, black line). Irradiation of 1·4H_2_O with 638 nm light at 10 K leads to the immediate drop of *χT* due to the sample heating with light, which is followed by a small increase and saturation after 60 minutes (Fig. S24†). The *χT* product at 10 K changes from 3.64 cm^3^ K mol^−1^ before irradiation to 4.57 cm^3^ K mol^−1^ immediately after turning the light off, which drops to 4.48 cm^3^ K mol^−1^ in 15 minutes. The same as 1, this experiment was followed by cooling to 2 K and *χT* measurement with 0.3 K min^−1^ heating rate (Fig. 7b, red line). The maximal observed value of 5.04 cm^3^ K mol^−1^ at 35 K is well below the level of 7.16 cm^3^ K mol^−1^ for the full HS phase. This makes LIESST for 1·4H_2_O quite inefficient, in line with previous conclusions on rigidity and slow dynamics of the framework at low temperature. Accordingly, the reverse-LIESST effect with 808 nm light is more effective with the photo-induced *χT* decreasing from 4.53 to 3.85 cm^3^ K mol^−1^, which accounts for ≈76% metastable HS centers (Fig. S25† and 7b, grey line). Surprisingly, *T*_LIESST_ determined for 1·4H_2_O after 638 nm irradiation equals 55 K, very similar to 1 (Fig. S26†). This may be correlated with the presence of the LIESST effect only for those centers in the structure of 1·4H_2_O that undergo a spin transition in the temperature range close to *T*_SCO_ in 1 (below 140 K). All the temperature-, light- and sorption-induced changes in the spin state of 1 are summarized in Fig. S27.†

## Conclusions

In search of three-dimensional breathing spin cross-over frameworks, we have prepared a coordination polymer {[Fe^II^(4-CNpy)_4_]_2_[W^IV^(CN)_8_]}_*n*_ (1). It shows an unusually low temperature of spin crossover transition *T*_1/2_ = 93 K, resulting from a very small enthalpy change. Despite the low temperature of the structural transformation, the spin transition is complete and proceeds relatively fast (as compared to other compounds demonstrating low-temperature SCO). This results from flexibility of the framework, which is confirmed by the observation of a gate-opening behavior induced by H_2_O and CO_2_ molecules. To the best of our knowledge, the transition between 1·4H_2_O and 1 is the first example of the gate-closing process studied by single-crystal XRD for a framework with only cyanides playing the role of a bridging ligand (without organic linkers). The breathing behavior resulting from water adsorption leads to the appearance of a 48 K wide hysteresis loop in the case of 1·4H_2_O, unusual among octacyanidometallate-based SCO compounds. More importantly, the inclusion of CO_2_, which is assisted by an almost 15% volume expansion upon the transition from 1 to 1·4CO_2_, results in the stabilization of the high-spin state in the entire temperature range studied. This shows that the “internal pressure” effect of rigid gas molecules can induce spin change in a spin cross-over framework, even in the absence of strong intermolecular interactions.

## Experimental

Potassium octacyanotungstate(iv) dihydrate was obtained according to the reported procedure.^65^ All other reagents were supplied by Sigma-Aldrich. Gases used for adsorption measurements were supplied by Nippon Sanso Holdings Corporation (He > 99.99995%, CO_2_ > 99.995%).

### Synthesis of {[Fe^II^(4-CNpy)_4_]_2_[W^IV^(CN)_8_]·4H_2_O}_*n*_ (1·4H_2_O)

In the first vial, K_4_[W^IV^(CN)_8_]·2H_2_O (0.026 mmol, 15 mg) was dissolved in 8 mL H_2_O, and in the second vial 4-cyanopyridine (9.0 mmol, 936 mg), Mohr's salt (0.048 mmol, 19 mg) and ascorbic acid (0.011 mmol, 2 mg) were dissolved in 24 mL H_2_O with 15 minutes of sonication in the ultrasonic bath. Then, both solutions were heated for 30 minutes at 36 °C in a water bath. Afterwards, a solution of octacyanotungstate(iv) was dropwise added to the iron(ii) containing mixture with hand stirring. The resulting clear yellow mixture was left at 36 °C to crystalize. The red octahedral crystals that appeared after 48 hours were collected by filtration. Yield: 10 mg (30%). Purity was confirmed by elemental analysis and powder X-ray diffraction (Fig. S2†). Anal. calcd for crystal structure (C_56_H_40_Fe_2_N_24_O_4_W): C: 47.75%, N: 23.86%, H: 2.86%. Found: C: 48.72%, N: 24.38%, H: 2.57%. The discrepancy results from partial sample dehydration before the start of the measurement. Calcd for C_56_H_37_Fe_2_N_24_O_2.5_W: C: 48.68%, C: 24.33%, H: 2.70%.

### Single crystal X-ray diffraction

Sc-XRD experiments for 1·4H_2_O and 1 were performed using a Bruker D8 Quest Eco diffractometer (Mo Kα sealed tube radiation source, Triumph® monochromator). Single crystals of 1·4H_2_O were moved directly from the mother liquor into NVH oil to avoid loss of the crystallization solvent. A single crystal of 1 was prepared *in situ* by heating 1·4H_2_O mounted on the goniometer head in a dry nitrogen stream using a Cryostream device (Oxford Cryosystems). Absorption corrections, data reduction and unit cell refinements were performed using SADABS and SAINT programs included in the Apex3 suite. The structures were solved using intrinsic phasing and refined anisotropically using weighted full-matrix least-squares on *F*^2^.^66–68^ Hydrogen atoms of the ligands were placed in calculated positions and refined as riding on the parent atoms. Structural diagrams were prepared using Mercury CSD 2020.3.0.^35^ CCDC 2240344 (1·4H_2_O at 200 K) and 2240345–2240347 (1 at 200, 140 and 80 K, respectively) contain the supplementary crystallographic data for this paper.

### Powder X-ray diffraction

PXRD data for phase purity confirmation (Fig. S2†) were obtained using a Bruker D8 Advance diffractometer (Cu Kα radiation) at room temperature for ground crystalline samples of 1·4H_2_O loaded into glass capillaries under mother liquor (0.7 mm in diameter). Sample of 1 was obtained by drying 1·4H_2_O in a vacuum (*p* ≈ 10^−2^ mbar) for 2 hours and inserted into a glass capillary in oxygen- and water-free atmosphere of a glovebox (O_2_ < 0.1 ppm, H_2_O < 0.5 ppm) and sealed with silicone grease. The results were subjected to background correction using the DIFFRAC algorithm implemented in the DIFFRAC.EVA V5 software. PXRD data for *in situ* CO_2_ adsorption measurement were collected for samples in 0.5 mm diameter glass capillaries using a Rigaku Ultima IV diffractometer (Cu Kα radiation). In order to allow vacuum treatment and CO_2_ introduction, the capillary was connected to stainless-steel (SUS) lines connected to a gas-handling system (BELSORP MAX; Microtrac BEL inc.). The temperature was controlled by a N_2_ gas stream. The results were background corrected by subtracting the pattern recorded for the empty sample stage at room temperature. The unit cell for 1·4CO_2_ was determined for the uncorrected measurement using FOX software,^69^ and Le Bail refinement was performed using JANA2006 software.^70^

### Volumetric adsorption measurements

The adsorption isotherm measurements were recorded on an automatic volumetric adsorption apparatus (BELSORP MAX; Microtrac BEL inc.) for *ca.* 25 mg sample of 1·4H_2_O activated by heating to 323 K under vacuum (*p* ≈ 10^−4^ mbar). For isotherm measurements of N_2_ and CO at 77 K and CO_2_ at 195 K, liquid nitrogen or dry ice/methanol bath was used. In the case of water vapor isotherm temperature was controlled using a water bath. For isobar measurements and isotherm measurements at different temperatures (including NO at 121 K), a home-built cryostat (ULVAC-Cryo) was utilized.

### Spectroscopic measurements

Infrared spectra for 1·4H_2_O were recorded using a Nicolet iN10 MX FT-IR microscope in transmission mode (a small amount of powdered sample was spread on a BaF_2_ pellet). Sample of 1 was obtained by *in situ* dehydration of 1·4H_2_O with dry nitrogen purge inside a Linkam THMS350V temperature-controlled stage. *In situ* CO_2_ adsorption was studied using a JASCO FT/IR-4200 spectrometer for the sample of 1 mixed with KBr and dispersed in between two CaF_2_ windows inside the cryostat (RC102, CRYO industries) connected to a gas-handling and pressure monitoring system (BELSORP MAX, Microtrac BEL inc.). Raman spectra during *in situ* CO_2_ adsorption were obtained using a JASCO NRS-4500 Raman microscope using a cryostat system (RC102, CRYO Industries) connected with a gas-handling and pressure-monitoring system (BELSORP MAX, Microtrac BEL inc.). Quartz and CaF_2_ were equipped over the sample room and the vacuum-insulating shield of the cryostat, respectively. The sample was inspected using an objective lens (Olympus SLMPLN20x) and was irradiated with a 532 nm Raman excitation laser. The transmission ^57^Fe Mössbauer spectra were collected using a Wissel spectrometer with a liquid nitrogen bath cryostat. A polycrystalline sample of 1·4H_2_O (*ca.* 50 mg) was inserted into a polyethylene (PE) bag with mother liquor to prevent its dehydration and sealed using an impulse heat sealer. Sample of 1 (*ca.* 50 mg) was obtained by drying 1·4H_2_O in a vacuum (*p* ≈ 10^−2^ mbar) for 2 hours, and then sealed inside a PE bag in an oxygen- and water-free atmosphere of a glovebox (O_2_ < 0.1 ppm, H_2_O < 0.5 ppm). The samples were mounted for the measurements inside a copper ring. Mössbauer spectra were fitted with the use of the WinNormos-for-Igor software package, assuming the Lorentzian shape of the resonance lines. In the case of 1·4H_2_O, two additional doublets (with a large value of quadrupole splitting) originating from the iron species in solution were included to improve the quality of the fits. Those signals (marked with purple asterisks in Fig. S5†) were excluded from the calculation of the relative fractions of different iron(ii) forms in the solid, which were determined from the ratio of the areas of the corresponding doublets attributed to 1·4H_2_O.

### Magnetic and photomagnetic measurements

Magnetic and photomagnetic studies for 1 and 1·4H_2_O were performed using a Quantum Design MPMS-3 Evercool magnetometer in magnetic fields up to 7 T. Polycrystalline sample of 1·4H_2_O (10.9 mg) was inserted into a polyethylene (PE) bag with a minimal amount of mother liquor and sealed using an impulse heat sealer. Sample of 1 (8.6 mg) was obtained by drying 1·4H_2_O in a vacuum (*p* ≈ 10^−2^ mbar) for 2 hours, and then sealed inside a PE bag in oxygen- and water-free atmosphere of a glovebox (O_2_ < 0.1 ppm, H_2_O < 0.5 ppm). Both samples were mounted onto the quartz sample holder using Kapton tape. The experimental data were corrected for the diamagnetism of the sample. Samples were prepared for photomagnetic measurements in the PE bags in a similar manner, but only a small amount (*ca.* 1 mg) was used, and PE bags were placed between two layers of adhesive tape (5 mm diameter) and inserted into the plastic straw. Irradiation was performed using laser diodes (power at the sample position 10–20 mW cm^−2^). Diamagnetic corrections were determined by comparison with bulk measurements.

### *In situ* CO_2_ adsorption magnetic measurements

Magnetic susceptibility measurements under a CO_2_ atmosphere were performed using a Quantum Design MPMS-XL on a polycrystalline sample of 1·4H_2_O (14.9 mg), which was placed inside a gelatin capsule with *ca.* 100 mg of polyester wool to ensure its immobilization. The capsule was pierced with a needle and placed inside a straw, which was attached to the home-built SUS (stainless steel) sample rod described elsewhere.^18^ The SUS tube was connected to the gas-handling system (BELSORP MAX; Microtrac BEL inc.). The sample was activated *in situ* by heating it to 323 K and vacuum pumping (*p* ≈ 10^−4^ mbar). Measurements for the sample in the activated state (1) were performed under 100 kPa He to ensure good thermal contact of the sample with the magnetometer cavity. Measurements for 1 under a CO_2_ atmosphere were performed with an additional 5 kPa He in the system, in order to preserve thermal conductivity after complete CO_2_ resublimation. The connection of the SUS sample rod to the gas-handling system was left open for the entire measurement time in order to enable constant monitoring of the gas pressure in the system. All the measurements were conducted in the temperature stabilization mode. Diamagnetic corrections were determined by comparison of the measurement for activated 1 with the bulk measurement for 1 in the PE bag.

### Additional measurements

TGA was performed using a NETZSCH TG 209 F1 Libra under a flow of nitrogen (20 mL min^−1^) and a temperature scanning rate of 2 °C min^−1^. DSC was performed using a NETZSCH DSC214 Polyma for the sample in a closed-lid sample holder at a temperature sweep rate of 5 K min^−1^. Elemental analyses were performed using an ELEMENTAR Vario Micro Cube CHNS analyzer.

## Data availability

Crystallographic data can be obtained *via*https://www.ccdc.cam.ac.uk/data_request/cif, or by emailing data_request@ccdc.cam.ac.uk, or by contacting The Cambridge Crystallographic Data Centre, 12 Union Road, Cambridge CB2 1EZ, UK; fax: +44 1223 336033. Data for figures presented in the article are available *via* the RODBUK repository https://rodbuk.pl/ and can be accessed directly through the following link: https://doi.org/10.57903/UJ/6SDMHB.

## Author contributions

M. Magott: conceptualization, funding acquisition, investigation and formal analysis (single crystal X-ray diffraction, magnetic and photomagnetic measurements, gas adsorption studies, spectroscopic and calorimetric measurements), supervision, visualization, writing – original draft, writing – review & editing. K. Płonka: investigation and formal analysis (synthesis, powder X-ray diffraction, TGA, spectroscopic measurements), writing – original draft. B. Sieklucka: funding acquisition, project administration, writing – review & editing. K. Dziedzic-Kocurek: investigation and formal analysis (Mössbauer spectroscopy). W. Kosaka: funding acquisition, investigation (gas adsorption and magnetic measurements), methodology (*in situ* CO_2_ adsorption studies), supervision, writing – review & editing. H. Miyasaka: funding acquisition, methodology (*in situ* CO_2_ adsorption studies), project administration, writing – review & editing. D. Pinkowicz: conceptualization, funding acquisition, investigation and formal analysis, project administration, writing – review & editing.

## Conflicts of interest

There are no conflicts to declare.

## Supplementary Material

SC-014-D3SC03255H-s001

SC-014-D3SC03255H-s002
