# Supplementary material for: Guest-induced pore breathing controls the spin state in a cyanido-bridged framework
Source: Chem Sci. 2023 Aug 16;14(36):9651–63. doi: 10.1039/d3sc03255h (PMC10510767; doi:10.1039/d3sc03255h)
Supplement: SC-014-D3SC03255H-s001 [file SC-014-D3SC03255H-s001.pdf]

Electronic Supplementary Material (ESI) for Chemical Science.
This journal is © The Royal Society of Chemistry 2023

Electronic Supplementary information to:

Guest-Induced Pore Breathing Controls Spin State in a Cyanido-Bridged Framework

Michał Magott,[*ab†] Klaudia Płonka,[a†] Barbara Sieklucka,[a] Katarzyna Dziedzic-Kocurek,[c] Wataru Kosaka,[b] Hitoshi Miyasaka,[*b] Dawid Pinkowicz[*a]

[a] Faculty of Chemistry, Jagiellonian University, Gronostajowa 2, 30-387 Kraków, Poland

[b] Institute for Materials Research, Tohoku University, 2-1-1 Katahira, Aoba-ku, Sendai 980-8577, Japan

[c] Marian Smoluchowski Institute of Physics, Jagiellonian University, Stanisława Łojasiewicza 11, Kraków 30-348, Poland

Table S1. Crystallographic data obtained from SCXRD for **1·4H$_2$O** and **1**.

	**1·4H$_2$O** (200 K)	**1** (200 K)	**1** (140 K)	**1** (80 K)
CCDC	2240344	2240345	2240346	2240347
Formula	$C_{56}H_{40}Fe_2N_{24}O_4W$	$C_{56}H_{32}Fe_2N_{24}W$		
FW / g mol^{-1}	1408.67	1336.60		
Crystal system	Tetragonal	Tetragonal		
Space group	$I4_1/a$	$I\bar{4}2d$ (refined as an inversion twin)		
a = b / Å	19.7947(3)	19.1966(4)	19.1481(4)	18.8695(4)
c / Å	16.0477(3)	15.8129(5)	15.7535(6)	15.4503(5)
V / Å^3	6288.0(2)	5827.2(3)	5776.0(3)	5501.2(3)
Z	4	4	4	4
ρ_{calc} / g cm^3	1.488	1.524	1.537	1.614
Abs. coeff. / mm^{-1}	2.34	2.52	2.54	2.67
F(000)	2808	2648		
Radiation source	Mo Kα	Mo Kα		
Crystal size / mm	0.24 x 0.10 x 0.08	0.12 x 0.11 x 0.06		
ϑ range / °	2.6-28.2	2.7-26.0	2.7-26.0	2.8-28.3
R_{int}	0.027	0.050	0.051	0.063
Parameters/restraints	204/3	193/3	193/0	193/0
GOF on F^2	1.10	1.14	1.16	1.11
R_1 (refl. with I > 2σ(I))	0.020	0.039	0.038	0.034
wR_2 (all reflections)	0.054	0.075	0.079	0.067
Largest diff. peak and hole / e Å^{-3}	0.75/-0.41	1.49/-2.49	1.34/-2.66	1.74/-2.46

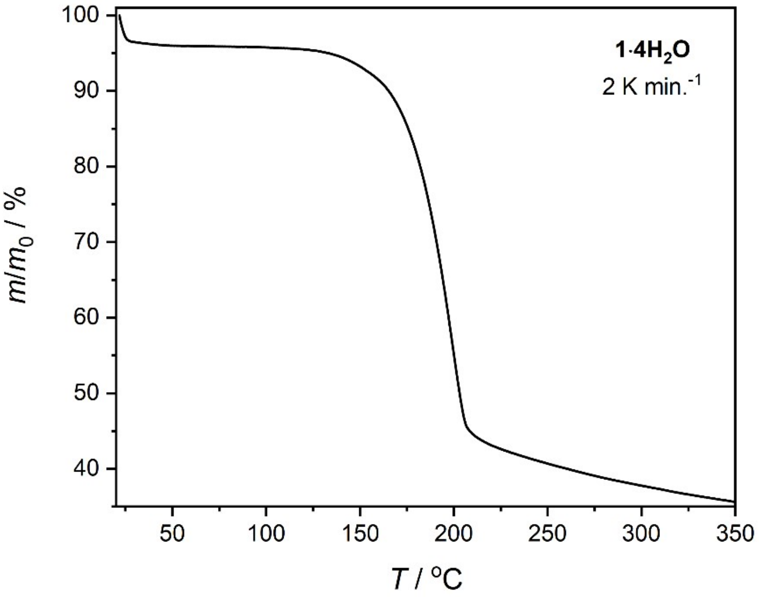

Figure S1. Thermogravimetric analysis of **1·4H$_2$O** recorded at a heating rate of 2 K min.$^{-1}$ under a dry nitrogen atmosphere.

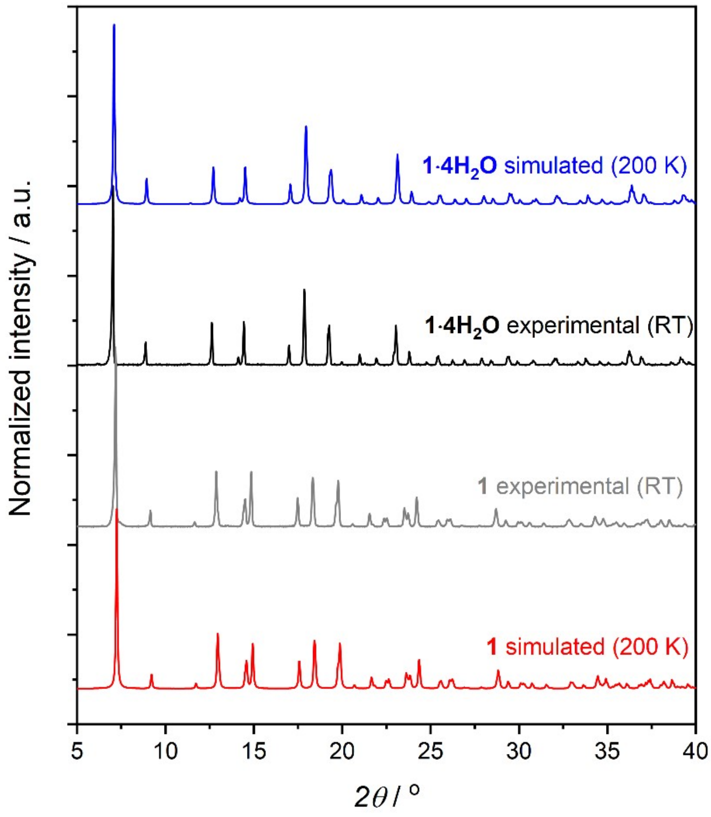

Figure S2. Powder X-ray diffraction pattern simulated from a single-crystal structure for **1·4H$_2$O** (blue line), experimental pattern for **1·4H$_2$O** (black line), experimental pattern for **1** (**1·4H$_2$O** activated at room temperature in vacuum – $p \approx 10^{-2}$ mbar; gray line) and simulated from a single-crystal structure for **1** (red line).

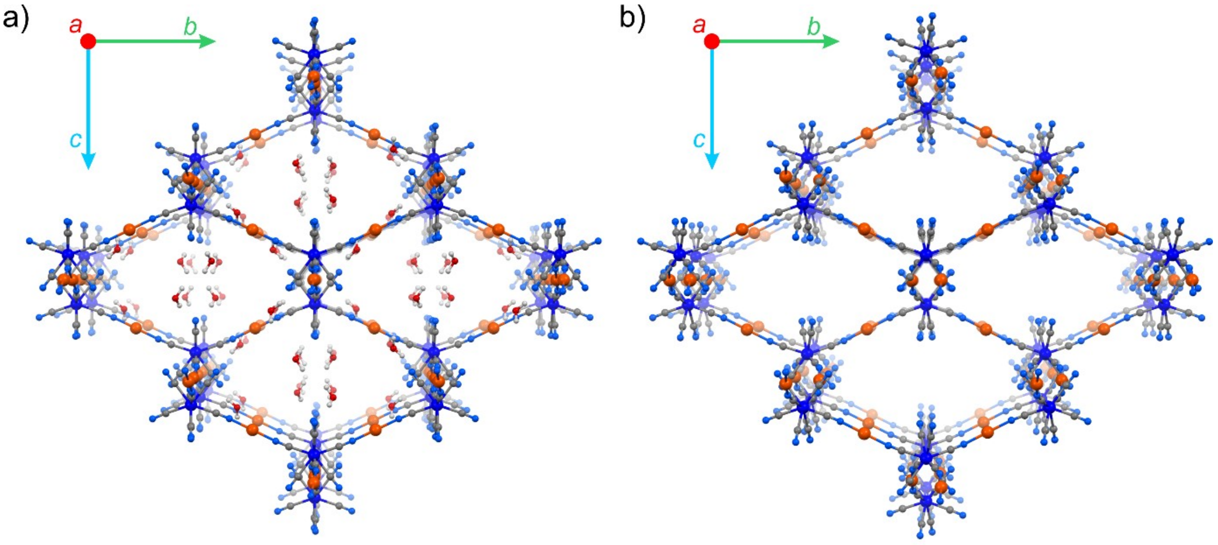

$1{\cdot}4H_2O$ **1**

Figure S3. Crystal structures of **$1{\cdot}4H_2O$** (a) and **1** (b) as seen along the crystallographic *a*-axis. 4-cyanopyridine molecules were omitted for clarity; W – dark blue, Fe – orange, O – red, N – blue, C – gray.

Table S2. Selected distances and bond length in structure of **$1{\cdot}4H_2O$** and **1**.

		**$1{\cdot}4H_2O$** (200 K)	**1** (200 K)	**1** (140 K)	**1** (80 K)
Bond lengths / Å	Fe-N_{CN}1	2.080(2)	2.085(7)	2.082(7)	1.958(5)
	Fe-N_{py}3	2.243(2)	2.207(13)	2.171(13)	2.016(9)
	Fe-N_{py}5	2.258(2)	2.252(7)	2.237(7)	2.070(6)
	Fe-N_{py}7	-	2.252(12)	2.251(13)	2.059(8)
Angles / °	N_{CN}1-Fe-N_{CN}1	180.0	175.8(4)	175.8(4)	174.2(3)
	N_{CN}1-Fe-N_{py}3	89.14(7)	87.9(2)	87.9(2)	87.1(2)
	N_{CN}1-Fe-N_{py}5	89.38(8)	89.3(2)	89.1(2)	89.7(2)
	N_{CN}1-Fe-N_{py}7	-	92.1(2)	92.1(2)	92.9(2)
	N_{py}3-Fe-N_{py}5	87.73(9)	90.0(2)	90.0(2)	90.6(1)
	N_{py}3-Fe-N_{py}7	-	180.0	180.0	180.0
	N_{py}5-Fe-N_{py}5	180.0	180.0(4)	179.9(4)	178.8(3)
	Fe-N_{CN}1-C_{CN}1	170.8(2)	162.4(7)	161.7(7)	165.2(5)
Intermolecular distances / Å	**hydrogen bond**				
	OH-N_{CN}	1.98(5)	-	-	-
	O-N_{CN}	2.867(4)	-	-	-
	OH-$N_{nitrile}$	2.30(5)	-	-	-
	O-$N_{nitrile}$	3.074(5)	-	-	-
	**hydrogen bond**				
	CH-N	2.695(4)	2.696(11)	2.661(10)	2.738(7)
	C-N	3.553(5)	3.43(2)	3.39(1)	3.44(1)
	**nitrile-nitrile**				
	N-C	3.244(6)	3.234(16)	3.224(12)	3.193(9)
		3.461(7)	3.362(10)	3.354(9)	3.296(6)

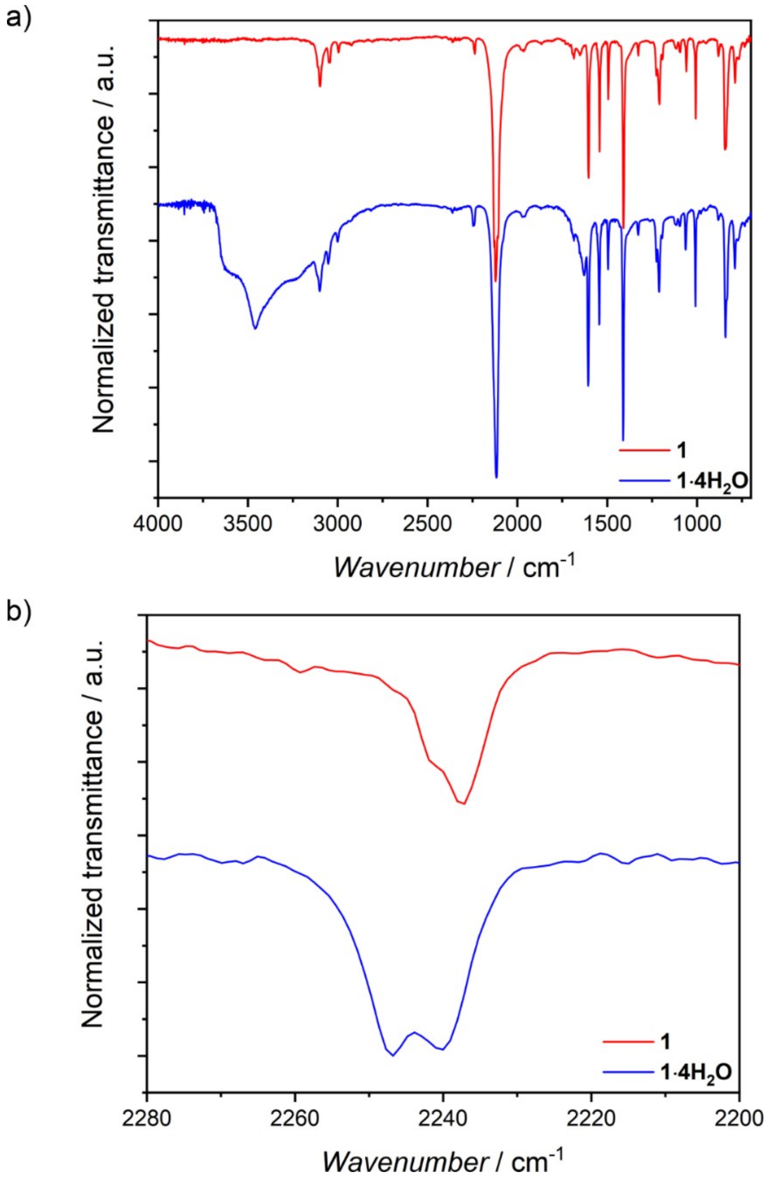

Figure S4. Infrared spectra of **1** and **1·4H$_2$O** recorded at room temperature using IR microscope in transmission mode (a) and close-up of the 2280-2000 cm^{-1} demonstrating the CN stretching band of 4-cyanopyridine ligands (b).

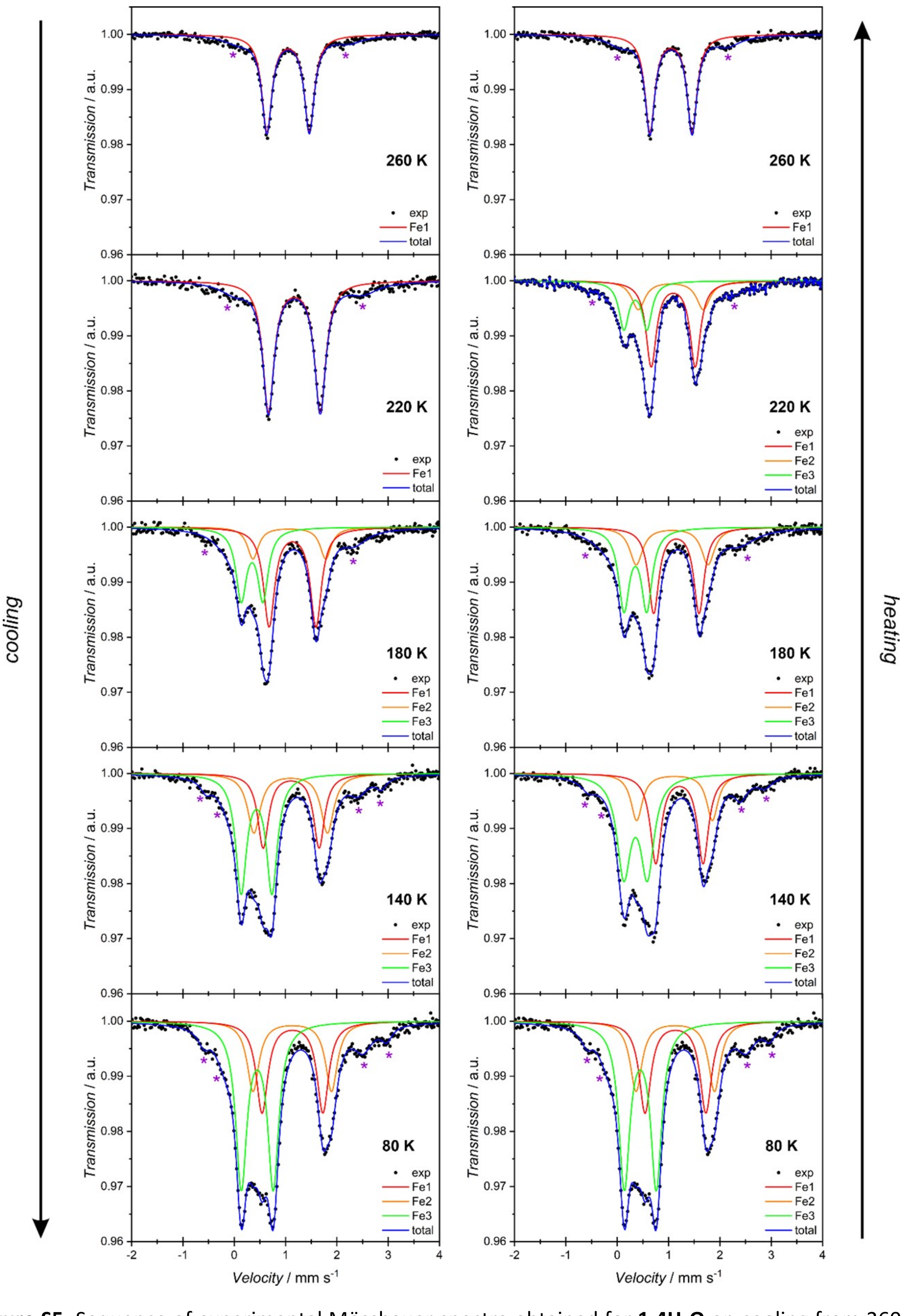

Figure S5. Sequence of experimental Mössbauer spectra obtained for **1·4H$_2$O** on cooling from 260 to 80 K and subsequent heating to 260 K (black points), and simulation (color lines). Purple asterisks denote small signals originating from the mother solution surrounding the solid. They were included in the fitting procedure, but the corresponding components were removed from Figure S5 for the sake of clarity.

Table S3. ^{57}Fe Mössbauer spectra parameters for **1·4H$_2$O**.

***T* / K**	**Fe site**	**δ_{IS} / mm·s^{-1}**	**ΔE_Q / mm·s^{-1}**	**fraction / %**
260(1) ↓	Fe1 (Fe^{II} HS)	1.05(1)	0.83(1)	100(1)
	Fe2 (Fe^{II} HS)	-	-	-
	Fe3 (Fe^{II} LS)	-	-	-
220(1) ↓	Fe1(Fe^{II} HS)	1.17(1)	1.01(1)	100(1)
	Fe2 (Fe^{II} HS)	-	-	-
	Fe3 (Fe^{II} LS)	-	-	-
180(1) ↓	Fe1 (Fe^{II} HS)	1.14(1)	0.91(1)	50(1)
	Fe2 (Fe^{II} HS)	1.08(1)	1.42(2)	16(1)
	Fe3 (Fe^{II} LS)	0.35(1)	0.42(1)	34(1)
140(1) ↓	Fe1 (Fe^{II} HS)	1.11(1)	1.09(1)	29(1)
	Fe2 (Fe^{II} HS)	1.10(1)	1.43(2)	26(1)
	Fe3 (Fe^{II} LS)	0.44(1)	0.60(1)	45(1)
80(1) ↓	Fe1 (Fe^{II} HS)	1.14(1)	1.19(1)	28(1)
	Fe2 (Fe^{II} HS)	1.13(1)	1.53(2)	23(1)
	Fe3 (Fe^{II} LS)	0.45(1)	0.62(1)	49(1)
140(1) ↑	Fe1 (Fe^{II} HS)	1.22(1)	0.92(1)	35(1)
	Fe2 (Fe^{II} HS)	1.12(1)	1.48(1)	19(1)
	Fe3 (Fe^{II} LS)	0.36(1)	0.46(3)	46(1)
180(1) ↑	Fe1 (Fe^{II} HS)	1.16(1)	0.89(1)	40(1)
	Fe2 (Fe^{II} HS)	1.07(1)	1.40(2)	22(1)
	Fe3 (Fe^{II} LS)	0.36(1)	0.44(1)	38(1)
220(1) ↑	Fe1 (Fe^{II} HS)	1.09(1)	0.86(1)	51(1)
	Fe2 (Fe^{II} HS)	1.05(1)	1.27(3)	22(1)
	Fe3 (Fe^{II} LS)	0.36(1)	0.45(1)	27(1)
260(1) ↑	Fe1 (Fe^{II} HS)	1.05(1)	0.82(1)	100(1)
	Fe2 (Fe^{II} HS)	-	-	-
	Fe3 (Fe^{II} LS)	-	-	-

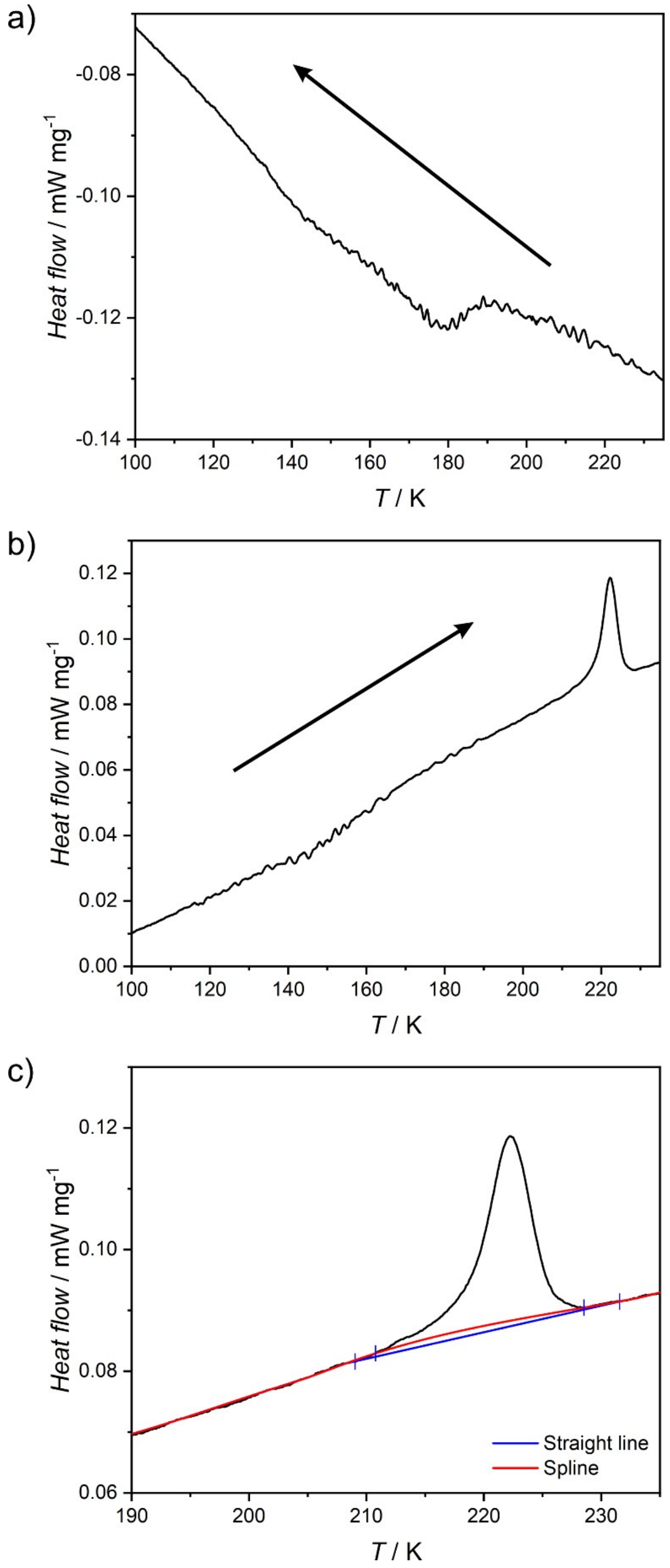

Figure S6. Dynamic scanning calorimetry (DSC) curves recorded at a scanning rate of 5 K min.$^{-1}$ on cooling (a) and heating (b) of **1·4H$_2$O**, as well as different types of background subtraction procedures used for determination of ΔH_{HL} value from the heating curve (c).

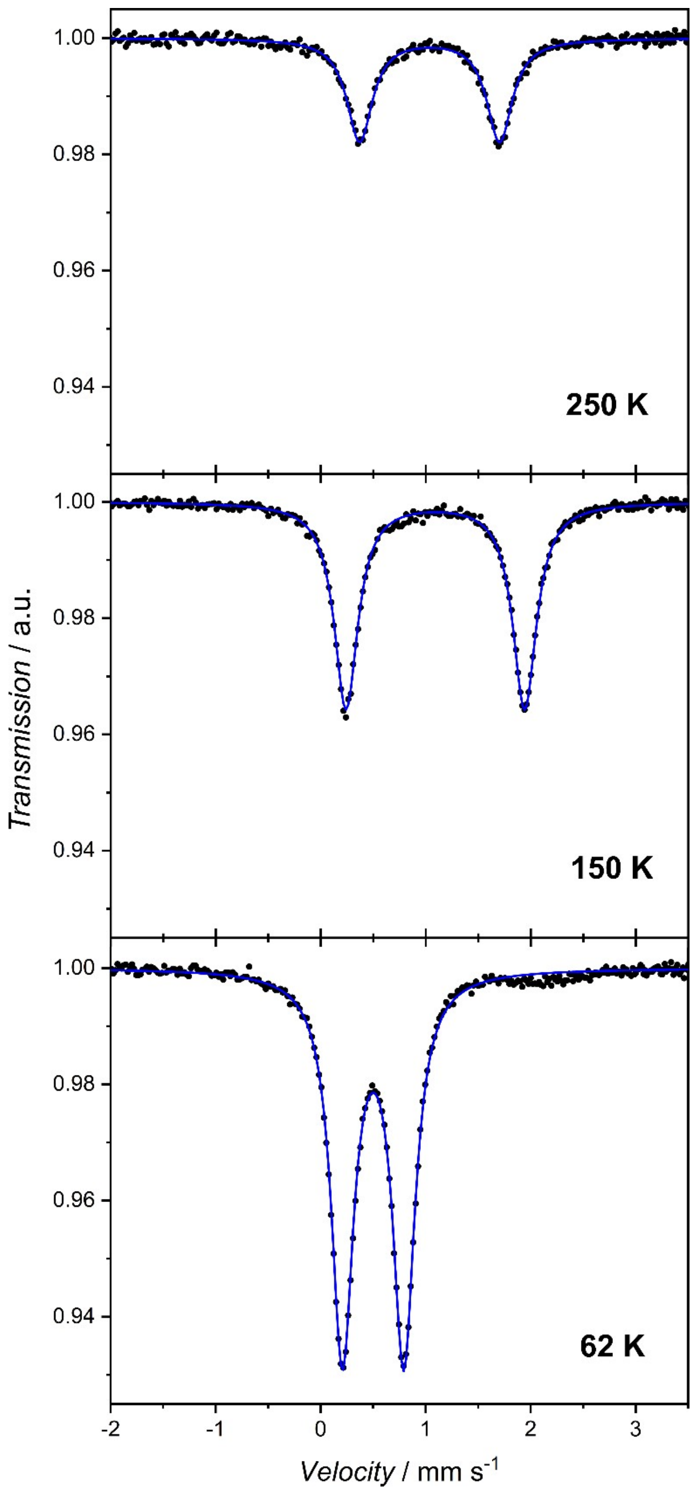

Figure S7. Mössbauer spectra obtained for **1** on cooling (black points) and single-component simulated signals (blue lines).

Table S4. ^{57}Fe Mössbauer spectra parameters for **1**.

***T* / K**	**Fe site**	**δ_{IS} / mm·s^{-1}**	**ΔE_Q / mm·s^{-1}**	**fraction / %**
250(1) ↓	Fe (HS)	1.04(1)	1.33(1)	100 %
	Fe (LS)	-	-	-
150(1) ↓	Fe (HS)	1.05(1)	0.82(1)	100 %
	Fe (LS)	-	-	-
62(1) ↓	Fe (HS)	-	-	-
	Fe (LS)	0.50(1)	0.56(1)	100 %

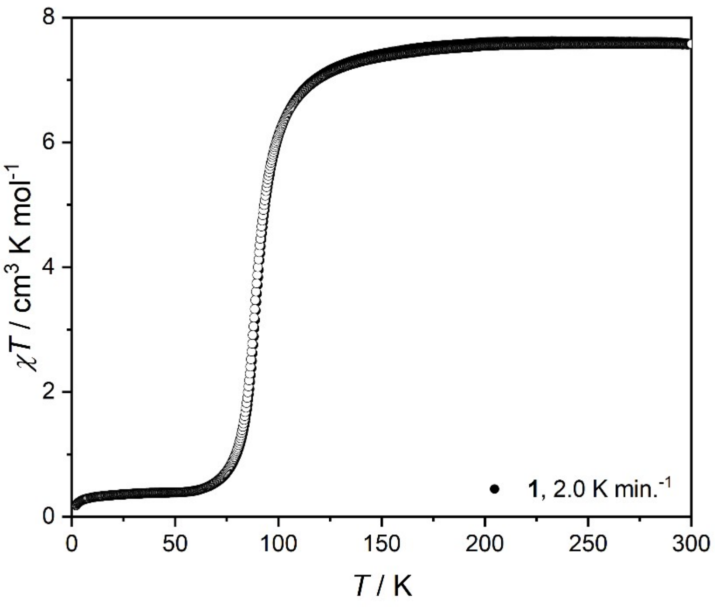

Figure S8. $\chi T(T)$ dependence recorded for **1** at 2 K min.$^{-1}$ on cooling (full circles) and heating (open circles) under $\mu_0 H_{dc}$ = 0.1 T.

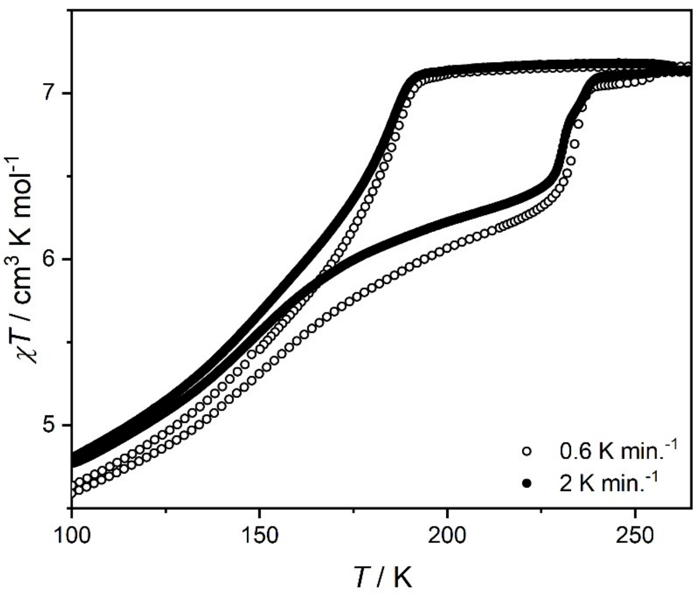

Figure S9. $\chi T(T)$ dependence recorded for **$1{\cdot}4H_2O$** at 0.6 K min.$^{-1}$ with temperature stabilized at each point (open circles) and with constant 2 K min.$^{-1}$ sweep (black points) under $\mu_0 H_{dc}$ = 0.1 T.

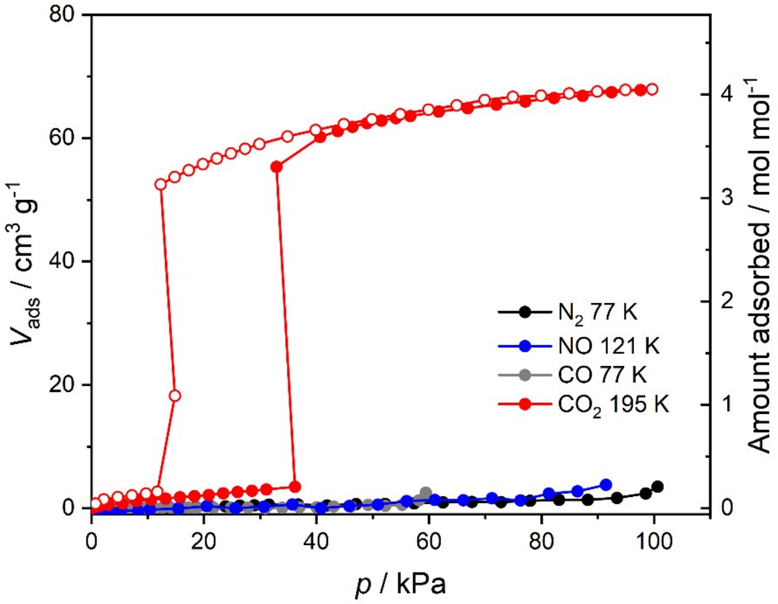

Figure S10. Volumetric measurement of gas adsorption for N_2, NO, CO and CO_2 in **1** (**$1{\cdot}4H_2O$** activated at 323 K). Full circles – adsorption, open circles – desorption. Solid lines are guides for the eye.

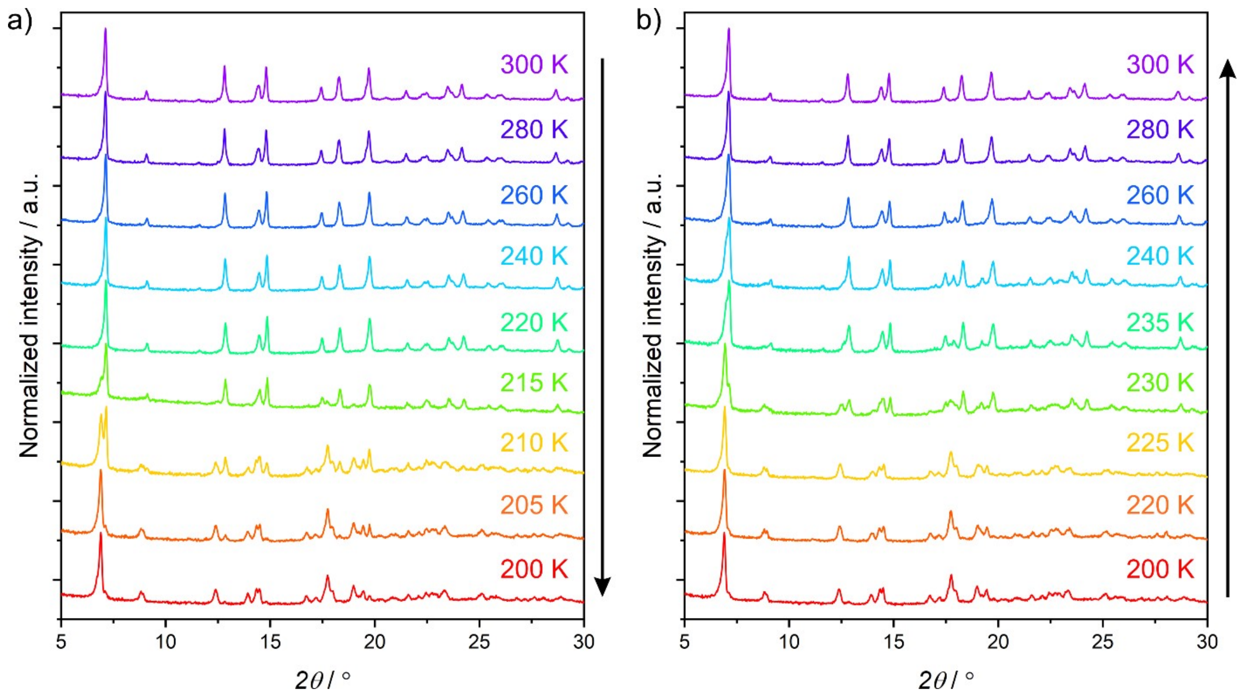

Figure S11. Powder X-ray diffraction patterns obtained for **1** under 100 kPa CO_2 atmosphere on cooling (a) and heating (b).

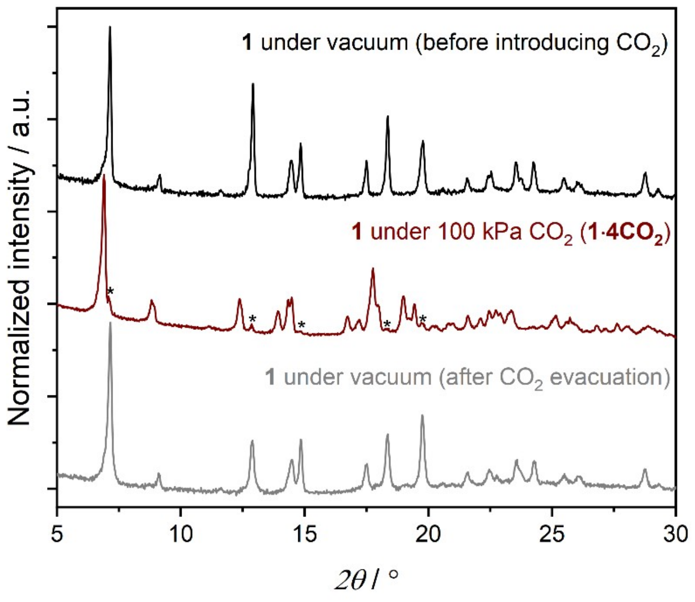

Figure S12. Powder X-ray diffraction patterns recorded for **1** at 200 K under vacuum (black line), under 100 kPa CO_2 atmosphere (dark red line), and after CO_2 removal (gray line). Asterisks denote the remnant reflections of phase **1** in **1·4CO₂**, which result from a small amount of powdered sample localized outside the cryocooler stream.

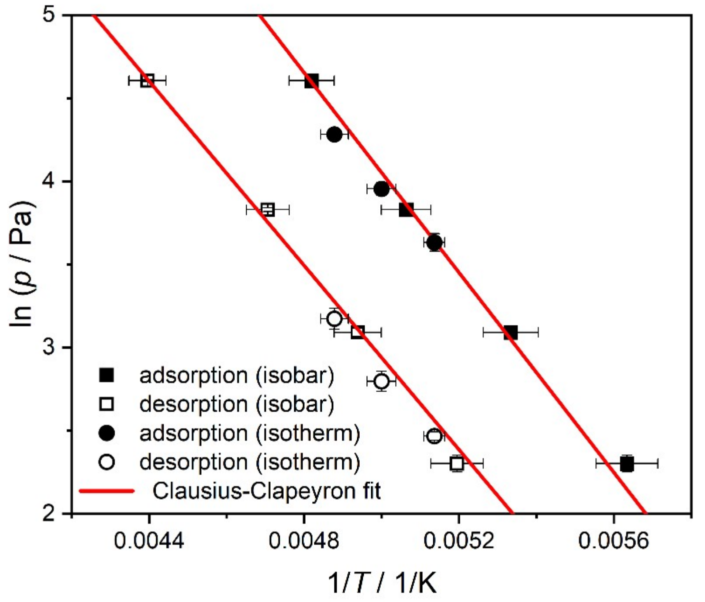

Figure S13. Clausius-Clapeyron fits of gate-opening (adsorption) and gate-closing (desorption) CO_2 pressure-temperature dependence for the transformation of **1** to **1·4CO₂** (and reverse).

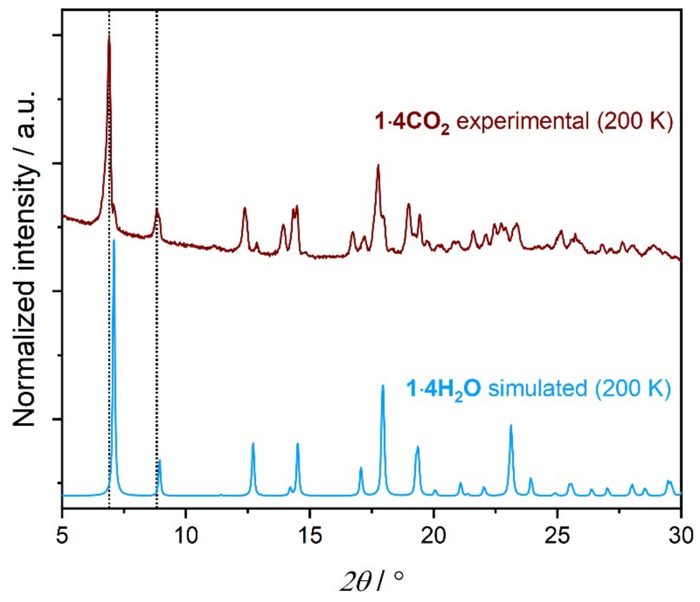

Figure S14. Comparison of the experimental PXRD pattern for **1·4CO₂** at 200 K (dark red line) and the simulated pattern for **1·4H₂O** at 200 K (blue line). The dotted lines show the shift in the position of the first two reflections, which in case of **1·4H₂O** correspond to the (101) and (200) Miller indices.

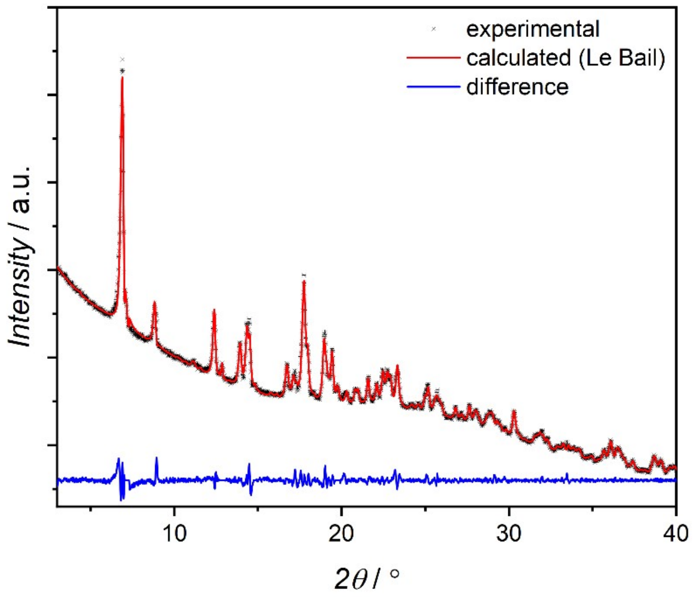

Figure S15. Comparison of the experimental PXRD pattern for **1·4CO$_2$** at 200 K (black points) and the Le Bail simulation for the orthorhombic unit cell (a = 20.364(3) Å, b = 19.950(3) Å, c = 16.461(3) Å and V = 6687(3) Å^3; red line), as well as the differential plot (I_c-I_o, blue line). The following regions containing reflections of phase **1** were excluded from simulation: [7.04, 7.30], [12.70, 13.00], [14.73,14.90] and [19.63,19.90].

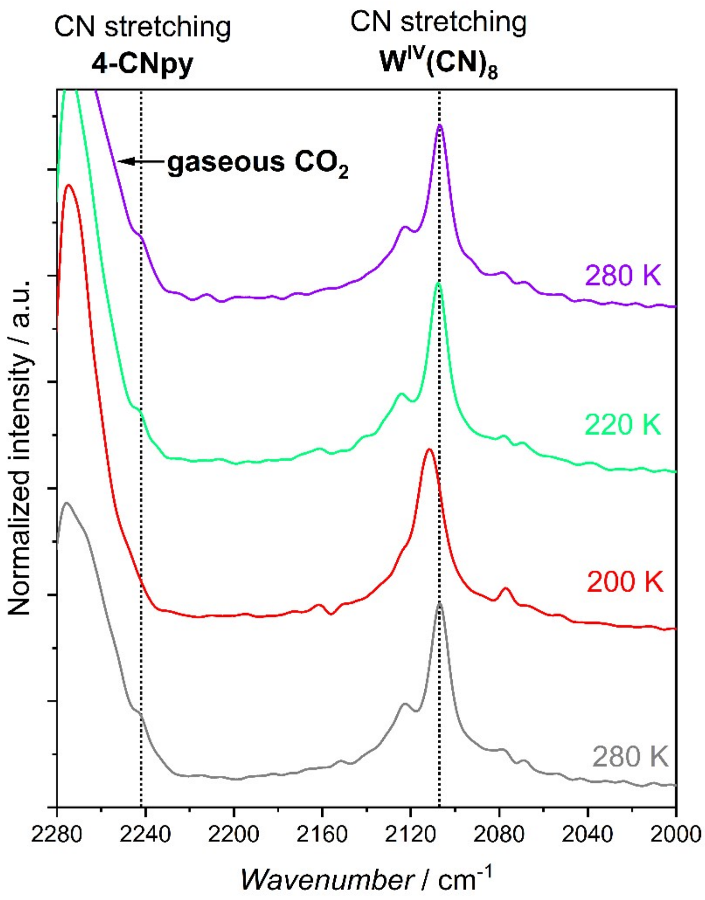

Figure S16. IR spectra recorded for **1** under 100 kPa CO_2 atmosphere at 280 K (purple line), 220 K (green line), 200 K (corresponding to the transition to **1·4CO$_2$**, red line) and after heating back to 280 K (which restores the initial **1**, gray line).

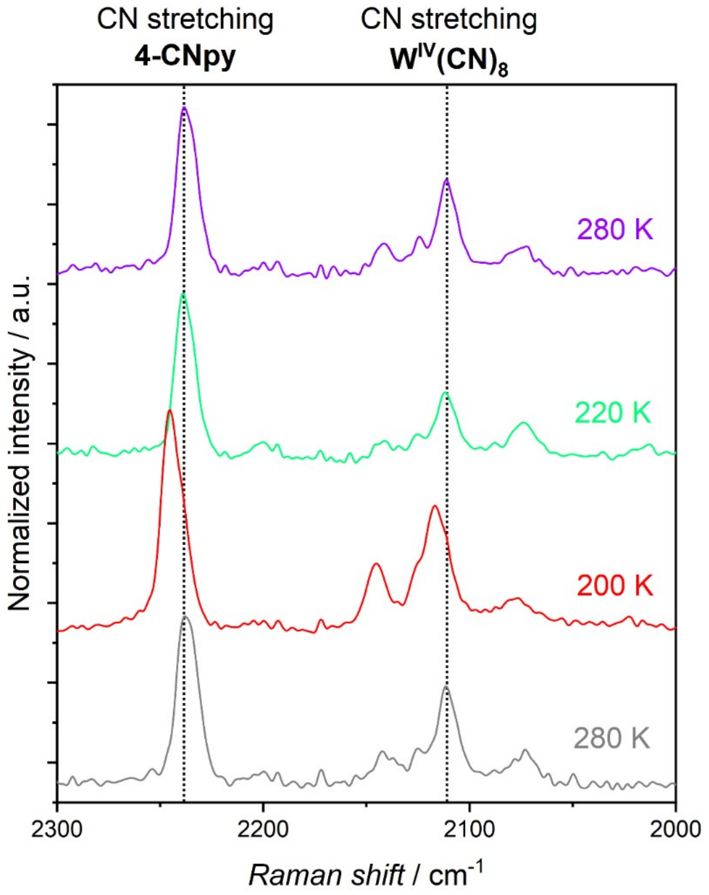

Figure S17. Raman spectra recorded for **1** under 100 kPa CO_2 atmosphere at 280 K (purple line), 220 K (green line), 200 K (corresponding to the transition to **1·4CO$_2$**, red line) and after heating back to 280 K (which restores the initial **1**, gray line).

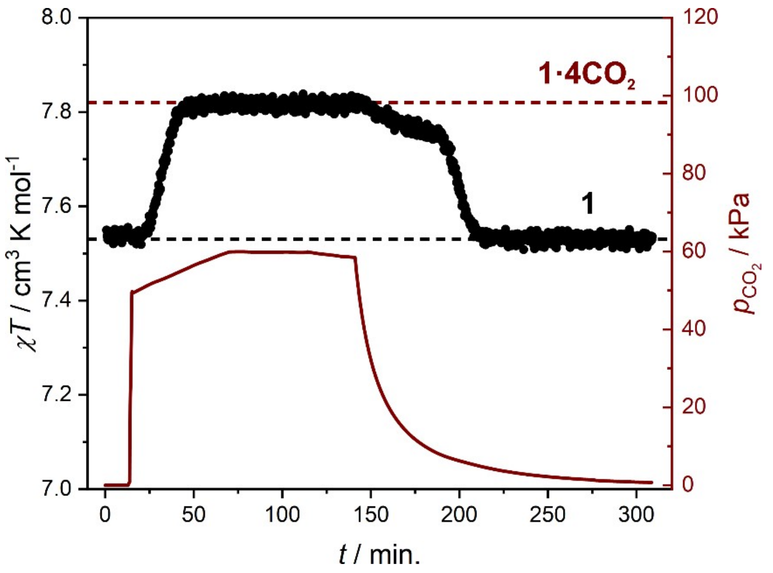

Figure S18. Time dependence of χT at $\mu_0 H_{dc}$ = 0.1 T (black line) and CO_2 pressure in the system (dark red line) during isothermal *in situ* CO_2 sorption experiment performed for **1** at 195 K.

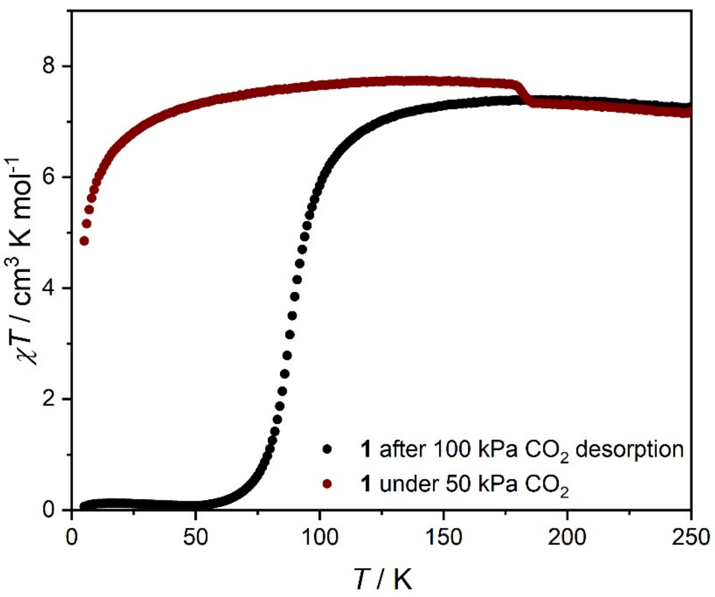

Figure S19. $\chi T(T)$ at $\mu_0 H_{dc}$ = 0.1 T recorded for **1** under 100 kPa He atmosphere (black line), after the *in situ* 100 kPa CO_2 adsorption experiment shown in the Figure 6, and the following measurement under 50 kPa CO_2 + 5 kPa He atmosphere (dark red line).

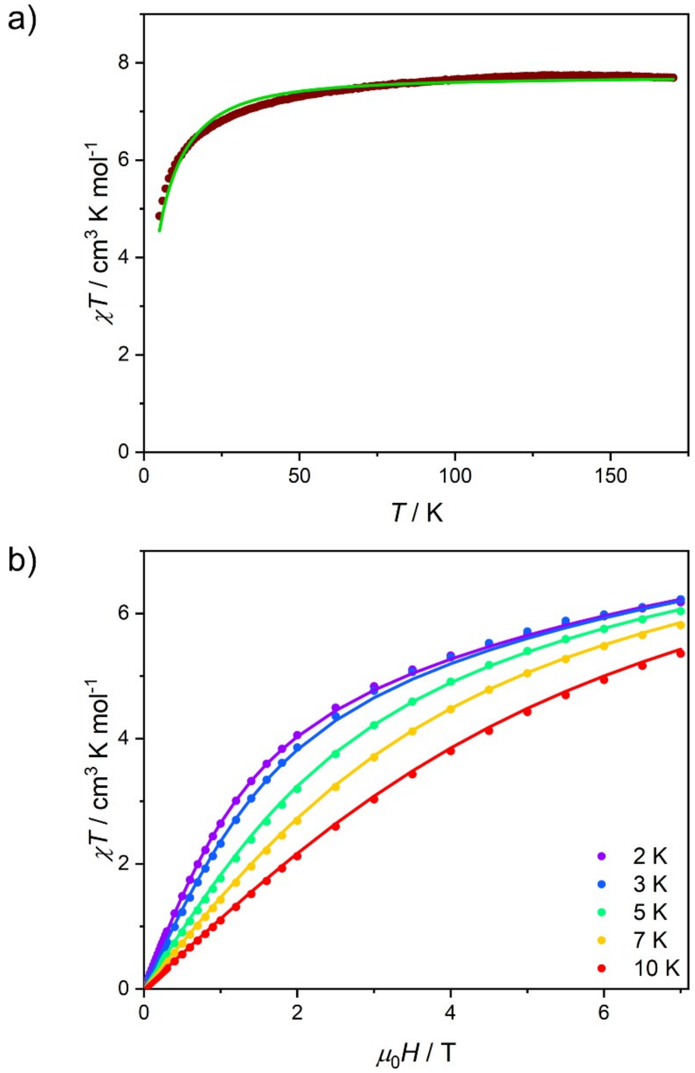

Figure S20. $\chi T(T)$ under $\mu_0 H_{dc}$ = 0.1 T (a) and $M(H)$ at T = 2-10 K (b) recorded for **1·4CO$_2$** (**1** under 50 kPa CO_2 atmosphere). Solid lines demonstrate the best fit to the equation 1 described in the main article text, which gives the following parameters: g = 2.27(5), D = 9.0(1) cm^{-1}, E = 2.3(1) cm^{-1} and zJ = -0.085(1) cm^{-1}.

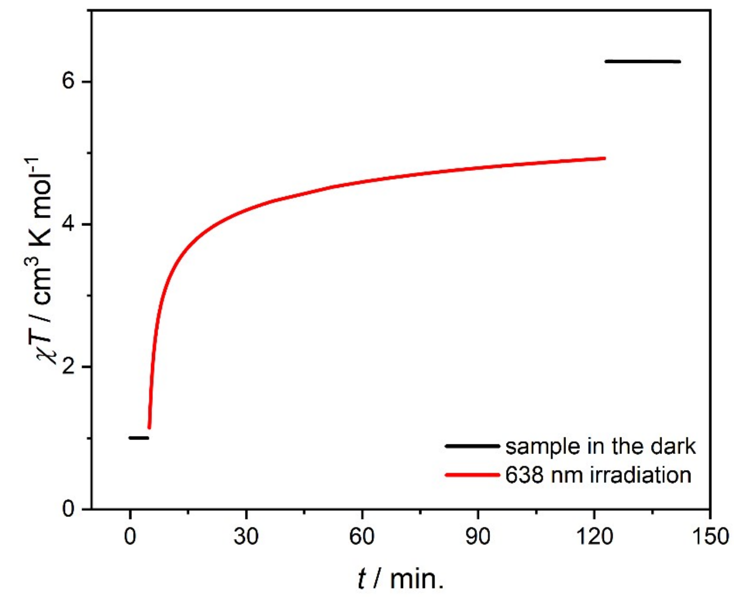

Figure S21. $\chi T(t)$ dependence recorded for **1** at $\mu_0 H_{dc}$ = 0.1 T during the 638 nm irradiation experiment.

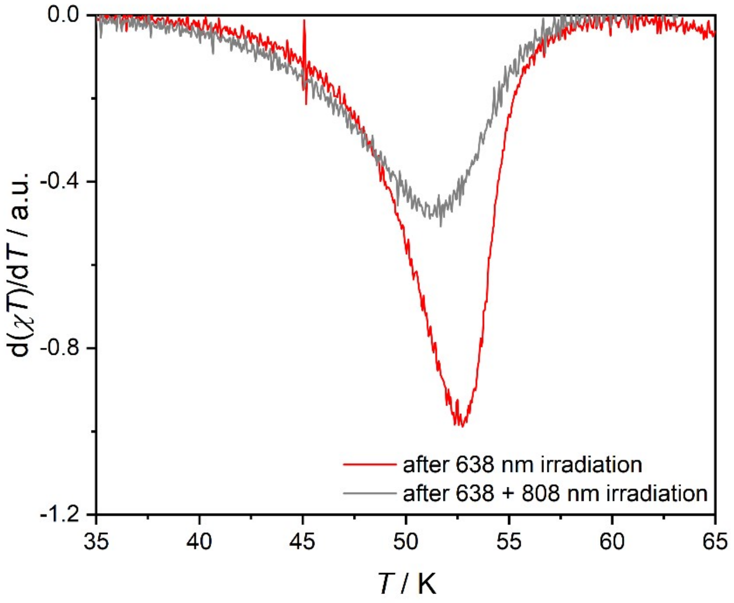

Figure S22. Derivative of $\chi T(T)$ curves for **1** after light irradiation (depicted in the Figure 4a).

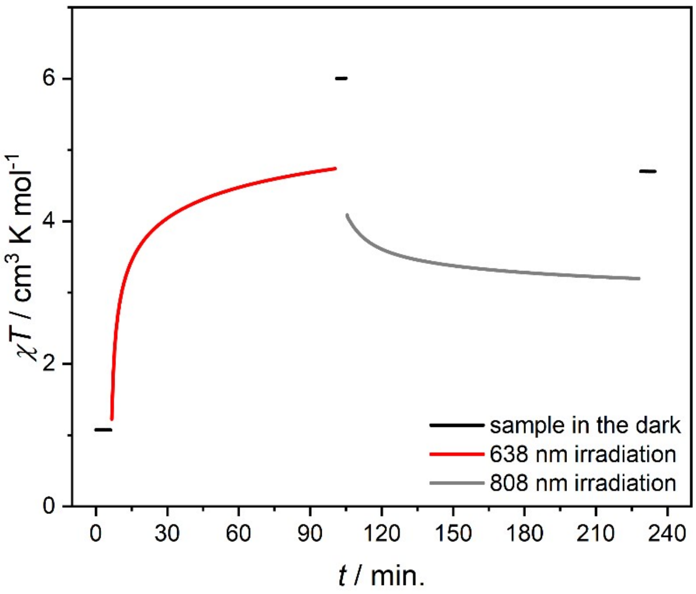

Figure S23. $\chi T(t)$ dependence recorded for **1** at μ_0H_{dc} = 0.1 T during the 638 and 808 nm irradiation experiment.

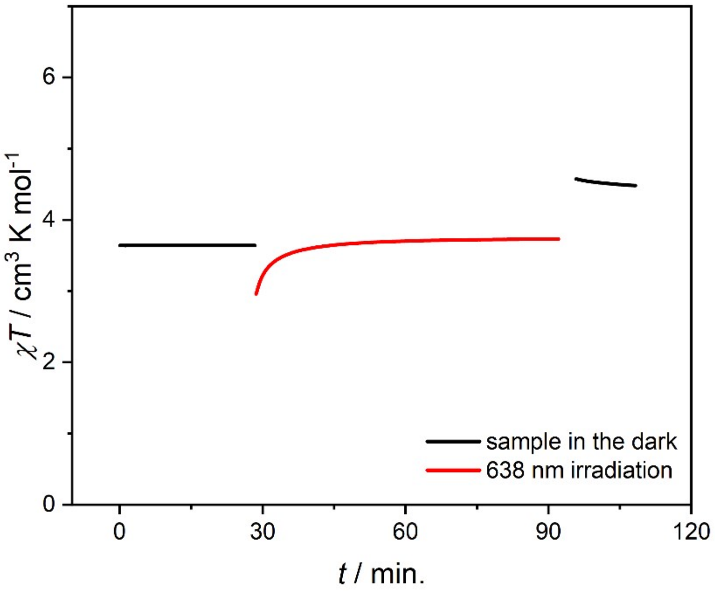

Figure S24. $\chi T(t)$ dependence recorded for **1·4H$_2$O** at μ_0H_{dc} = 0.1 T during the 638 nm irradiation experiment.

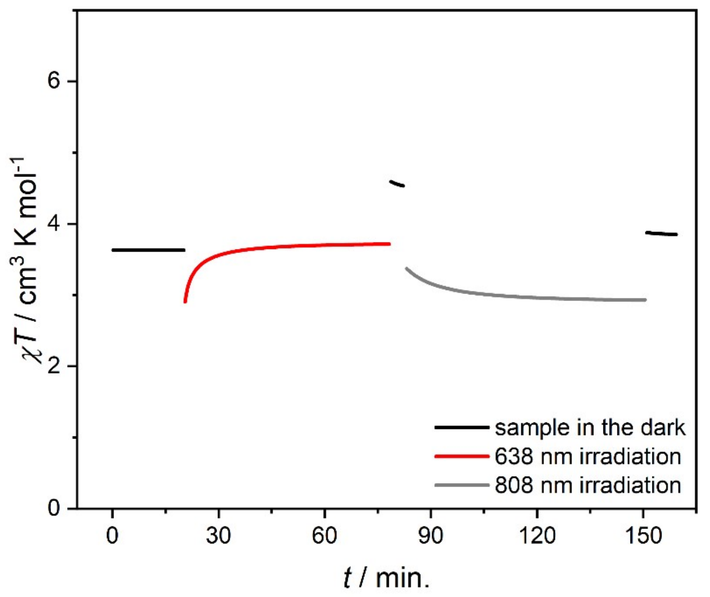

Figure S25. $\chi T(t)$ dependence recorded for **1·4H$_2$O** at $\mu_0 H_{dc}$ = 0.1 T during the 638 and 808 nm irradiation experiment.

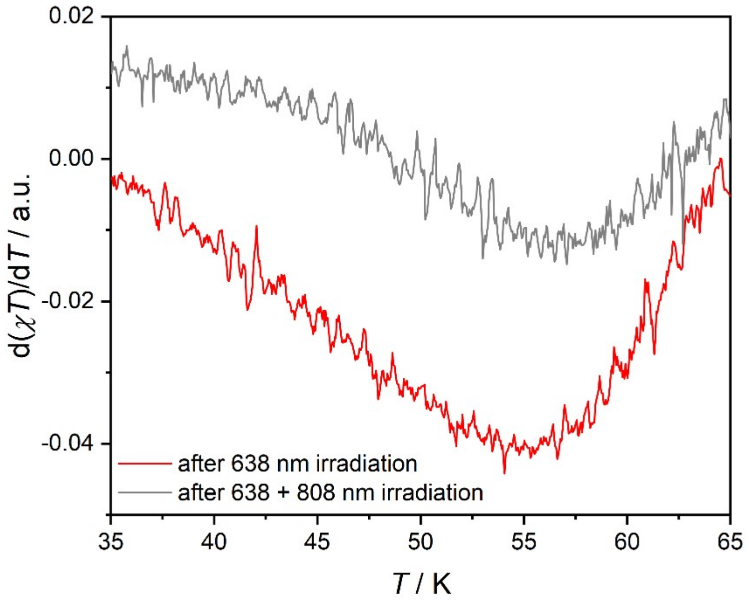

Figure S26. Derivative of $\chi T(T)$ curves for **1·4H$_2$O** after light irradiation (depicted in the Figure 4b).

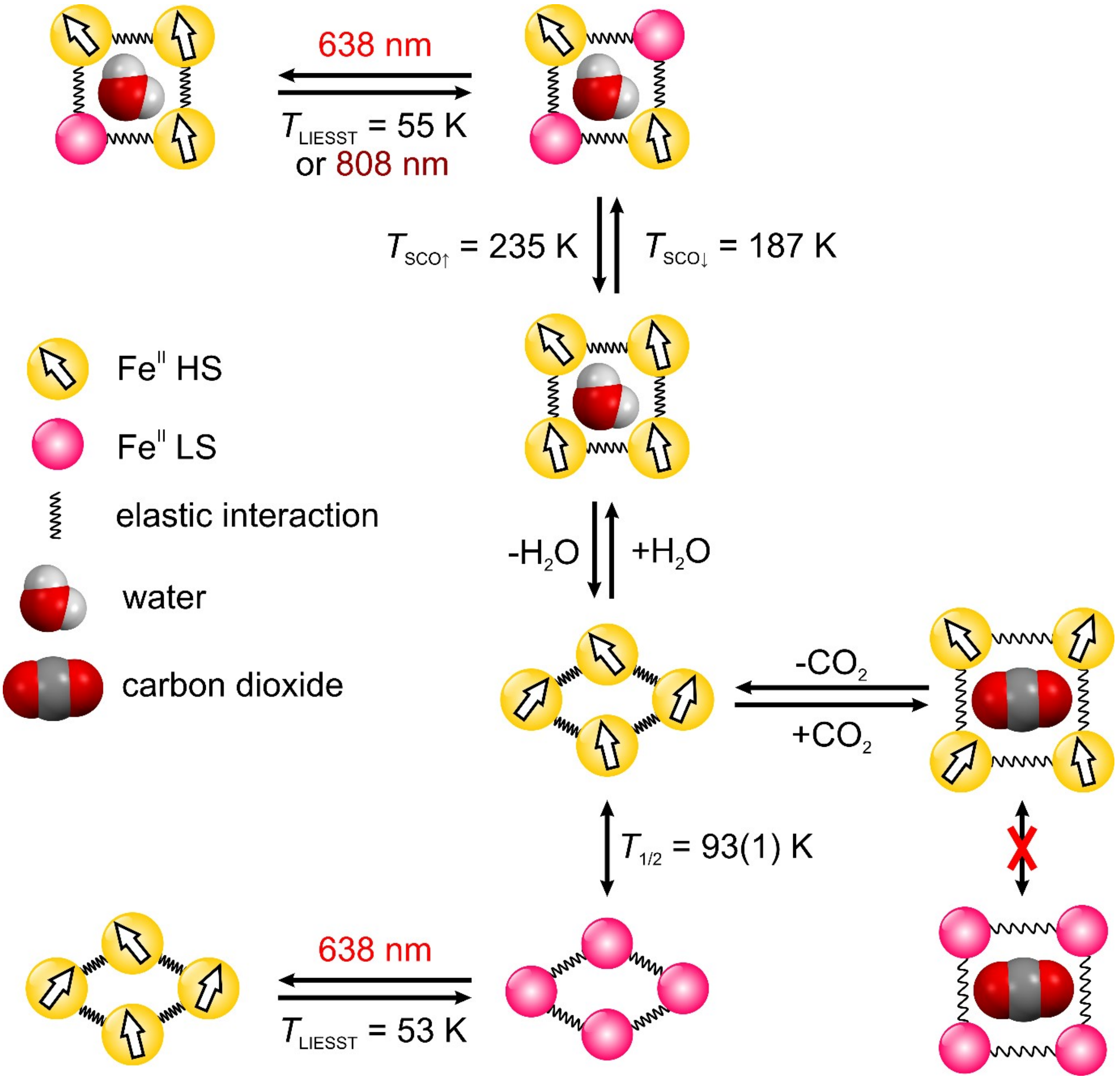

Figure S27. Schematic depiction of temperature-, light- and sorption-induced changes in the spin state of **1.**
